# High-performance Marangoni hydrogel rotors with asymmetric porosity and drag reduction profile

**DOI:** 10.1038/s41467-022-35186-5

**Published:** 2023-01-03

**Authors:** Hao Wu, Yiyu Chen, Wenlong Xu, Chen Xin, Tao Wu, Wei Feng, Hao Yu, Chao Chen, Shaojun Jiang, Yachao Zhang, Xiaojie Wang, Minghui Duan, Cong Zhang, Shunli Liu, Dawei Wang, Yanlei Hu, Jiawen Li, Erqiang Li, HengAn Wu, Jiaru Chu, Dong Wu

**Affiliations:** 1 CAS Key Laboratory of Mechanical Behavior and Design of Materials, Key Laboratory of Precision Scientific Instrumentation of Anhui Higher Education Institutes, Department of Precision Machinery and Precision Instrumentation, University of Science and Technology of China, Hefei, 230027 China; 2 Key Laboratory of Testing Technology for Manufacturing Process of Ministry of Education, Southwest University of Science and Technology, Mianyang, 621010 China; 3 Department of Modern Mechanics, University of Science and Technology of China, Hefei, 230026 China; 4 Physical Intelligence Department, Max Planck Institute for Intelligent Systems, 70569 Stuttgart, Germany

**Keywords:** Actuators, Mechanical engineering, Gels and hydrogels, Electrical and electronic engineering, Fluid dynamics

## Abstract

Miniaturized rotors based on Marangoni effect have attracted great attentions due to their promising applications in propulsion and power generation. Despite intensive studies, the development of Marangoni rotors with high rotation output and fuel economy remains challenging. To address this challenge, we introduce an asymmetric porosity strategy to fabricate Marangoni rotor composed of thermoresponsive hydrogel and low surface tension anesthetic metabolite. Combining enhanced Marangoni propulsion of asymmetric porosity with drag reduction of well-designed profile, our rotor precedes previous studies in rotation output (~15 times) and fuel economy (~34% higher). Utilizing thermoresponsive hydrogel, the rotor realizes rapid refueling within 33 s. Moreover, iron-powder dopant further imparts the rotors with individual-specific locomotion in group under magnetic stimuli. Significantly, diverse functionalities including kinetic energy transmission, mini-generator and environmental remediation are demonstrated, which open new perspectives for designing miniaturized rotating machineries and inspire researchers in robotics, energy, and environment.

## Abstract (web summary)

Utilizing the Marangoni effect, miniaturized machineries can be endowed with self-propulsion. Here, the authors report high-performance Marangoni hydrogel rotors with asymmetric porosity and drag reduction profile and demonstrate multiple potential functionalities.

## Introduction

Rotating machineries are commonly utilized in our daily life for various applications, such as power generation, liquid pumping, propulsion and ventilation^1^. With the demand for miniaturization of these functional machineries, the rotors integrated therein also need to be miniaturized while powering the miniaturized rotors has become a major challenge^2^. In recent years, different energy sources including sound^3,4^, light^5,6^, electricity^7,8^, and magnetism^9,10^ have been successfully converted into the kinetic energy of miniaturized rotors. However, external energy input is indispensable in the above-mentioned works, which impels researchers to develop self-propelled rotors.

**Fig. 1:**
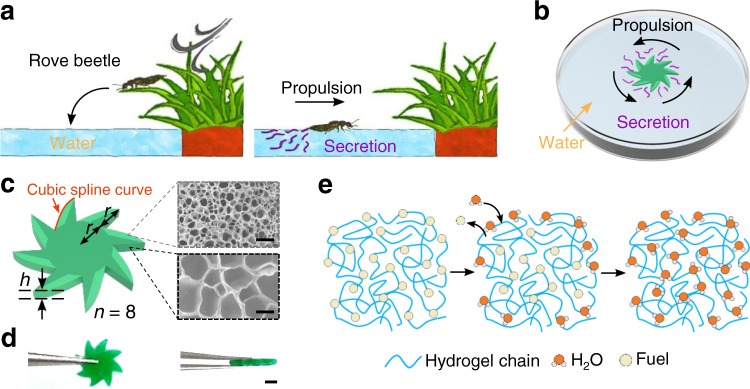
Propulsion mechanism of the rove beetle in genus *Stenus* and the bio-inspired Marangoni hydrogel rotor. **a** When a rove beetle accidentally falls on the water, it can secrete surface-active substances at its tail to quickly move to the shore. **b** Similar to the rove beetle, bio-inspired hydrogel rotor can secrete organic fuel to keep itself moving at the air-water interface. **c** The hydrogel rotor is defined by three geometric parameters: radius *r*, thickness *h* and teeth number *n*. The profile of rotor tooth is a well-designed cubic spline curve, and its control point coordinates are shown in Supplementary Fig. 3. Inserted are SEM images of the porous structures in different parts of hydrogel rotor. **d** Fabricated hydrogel rotor top-view and side-view images with characteristic geometric parameters: *r* = 1000 µm, *h* = 629 µm, and *n* = 8. **e** Propulsion mechanism: HFIP fuel is initially trapped in the hydrogel chain, and it is released to the surrounding medium when the rotor comes in contact with water. Trapped HFIP molecules are replaced by water molecules. The scale bars are 20 μm in (**c**), 1 mm in (**d**).

Self-propelled phenomena are ubiquitous in nature, ranging from nanometer scale (e.g., kinesin)^11^ to centimeter scale (e.g., arthropod)^12^. For instance, when a rove beetle in genus *Stenus* (a kind of insect living around ponds) accidentally falls on the water, it can secrete surface-active substances to propel itself back to the shore at several times its crawling speed (Fig. 1a)^13,14^. This self-propelled effect is caused by the surface tension gradient, known as Marangoni effect^15^. Marangoni effect has inspired the development of artificial self-propelled rotors, and various strategies have been explored for the design of Marangoni rotors based on porous materials (e.g., gels^16–22^, foams^23,24^ and metal-organic frameworks (MOFs)^25,26^). Different chemical fuels including ethanol^16,19,23^, tetrahydrofuran^17^, 1-Ethyl-3-methylimidazolium-bis(trifluoromethylsulfonyl)imide^18^, polyvinyl alcohol^20^, sodium dodecyl sulfonate^22^, camphor^24^, dimethylformamide^25^ and diphenylalanine peptide^26^ were encapsulated inside porous materials. Although these Marangoni rotors have been continuously optimized, there are still some major limitations: (a) lack of geometric and structural design (i.e., design does not consider drag reduction and enhancement of surface tension gradient), (b) low rotation output and fuel economy, and (c) incapable of individual-specific locomotion of rotor group. Furthermore, in order to pursue higher rotation speed, some Marangoni rotors are very thin (e.g., tens of microns)^16,25^ or made of materials with low stiffness (e.g., droplet rotors)^27,28^, which cannot be used for kinetic energy transmission and hinders practical applications.

Herein, we answer above challenges by proposing bio-inspired Marangoni rotor composed of poly(N-isopropylacrylamide) (PNIPAm) and hexafluoroisopropanol (HFIP, a metabolite of inhalation anesthetics). Due to the low surface tension of HFIP fuel (especially maintaining low surface tension at very low concentrations) and special rotor design (asymmetric porosity for the enhancement of surface tension gradient, well-designed shape for drag reduction), our hydrogel rotor has dominant advantages in comparison with traditional chemical Marangoni rotors: higher rotation output (up to 27175.15 rpm/mm^3^, ~15 times the best reported work) and fuel economy (up to 39.33 min/mg, ~34% higher than the best reported work)^17–19,25–35^ (Supplementary Table 4), rapid refueling (within 33 s, leveraging on thermoresponsive PNIPAm), individual-specific locomotion of hydrogel rotor group (e. g., three individual rotors can revolve simultaneously along different radii and directions). In addition, our hydrogel rotors exhibit high rotation position accuracy (<120 µm) due to high precision of grayscale digital light processing. More significantly, distinct functionalities based on our hydrogel rotor have been deployed, including kinetic energy transmission as speed reducer and multiplier, energy conversion as the mini-generator, and removal of heavy metal ions in water for environmental remediation.

## Results

### Propulsion mechanism of bio-inspired Marangoni hydrogel rotor

Similar to the rove beetle in genus *Stenus* which can secrete surfactants to move on the water surface, our bio-inspired hydrogel rotor can also secrete organic fuel to keep itself moving quickly at the air–water interface (Fig. 1b). The main frame of bio-inspired hydrogel rotor is composed of PNIPAm. In order to leverage the tunable physical and chemical properties of PNIPAm, a facile microfabrication process is developed to fabricate hydrogel rotors (Supplementary Fig. 1). Based on the grayscale digital light processing which uses digital micromirror device (DMD) (Supplementary Fig. 2), we can easily control the rotor shape and the microstructure in different parts of hydrogel rotor. For the rotor shape, we adopted a well-designed cubic spline curve (marked as Type-III_Sp_, Supplementary Fig. 3) to reduce drag, which is discussed in detail in the next section. For the microstructure in different parts of hydrogel rotor, we used exposure dose to control the crosslinking density of hydrogel. As shown in scanning electron microscope (SEM) images in Fig. 1c, the surface porosity on both sides of each tooth of the hydrogel rotor is asymmetric, with a surface porosity of 40.8 ± 1.7% in the light green part and 78.8 ± 2.0% in the dark green part (Supplementary Fig. 4). Asymmetric porosity affects the initial concentration and diffusion coefficient of the organic fuel, thereby enhancing the surface tension torque, which is also discussed in detail in the next section. The geometry of the rotor is mainly determined by the following parameters: radius *r*, thickness *h* and teeth number *n* (Fig. 1c). The thickness *h* was controlled by the volume of hydrogel precursor (Supplementary Fig. 5). According to the described design, we prepared different hydrogel rotors with radius *r* ranging from 250 to 1000 µm, thickness *h* ranging from 246 to 790 µm, teeth number *n* ranging from 2 to 8 (Fig. 1d and Supplementary Fig. 6).

After DMD exposure, the formed rotor was transferred to HFIP solvent for development and fuel filling. The uncured hydrogel was dissolved by HFIP, and a large amount of HFIP (~76.6% w/w, Supplementary Fig. 7) remained in the rotors due to the porous microstructure of hydrogel after curing (Fig. 1c). The HFIP in the rotor acts as fuel. In an aqueous environment, the water molecules around the rotor can replace the HFIP molecules trapped in the hydrogel chains^16–19,36^ (Fig. 1e). When the rotor is placed at the air-water interface, it can slowly secrete HFIP to form a surface tension gradient around itself and generate driving torque to drive itself to rotate rapidly^21,22,25^.

HFIP is chosen as rotor fuel for the following reasons. Firstly, HFIP is a metabolite of sevoflurane, which is approved by the Food and Drug Administration (FDA) and widely used in inhaled general anesthesia^37^. Therefore, HFIP has a well-documented pharmacokinetics (details in Supplementary Note 1). In addition, HFIP has great advantages over other common organic solvents due to its low surface tension, especially at very low concentrations^36^. For example, when HFIP concentration is 10% (v/v), the surface tension is still as low as ~22 mN/m (the surface tension of pure HFIP is 14.53 mN/m, for comparison) (Supplementary Fig. 8). Therefore, tiny HFIP fuel is capable of generating giant surface tension gradients, which can provide greater Marangoni propulsive forces than other common organic fuels. Moreover, the porous structure of the hydrogel and the capture of HFIP by the hydrogel chains guarantee high storage and slow release of fuel^16–19^. Choosing HFIP ensures that the bio-inspired hydrogel rotor has higher rotation speed and longer lifetime in comparison to those using other organic fuels (Supplementary Table 1). Due to the synergy of hydrogel-fuel, enhancement of surface tension torque by asymmetric porosity and reduction of drag by well-designed shape, the rotor exhibits excellent performance including high rotation speed and long lifetime. Besides, the self-propulsion of Marangoni hydrogel rotors in water is a great advantage compared with other chemical rotor systems which require acidic or other ionic solutions, hydrogen peroxide, or chemical reactions for rotation^38–41^. This expands the applicability of the bio-inspired hydrogel rotors and permits the utilization in biological applications^36^. To explore more forms of motion, we also fabricated a hydrogel disc with asymmetric porosity which can move in a straight line on the water surface (Supplementary Fig. 9).

### Enhancement of surface tension torque and shape design for drag reduction

The rotor can rotate due to the different surface tensions on both sides of each tooth, and the different surface tensions are caused by the different concentrations of HFIP fuel. During the rotation of the rotor, the torques created by surface tension and resistance compete with each other. The performance of the rotor can be increased by enhancing surface tension torque and reducing resistance torque. The research in this section is based on the characteristic rotor (*r* = 250 µm, *h* = 474 µm, and *n* = 8).

**Fig. 2:**
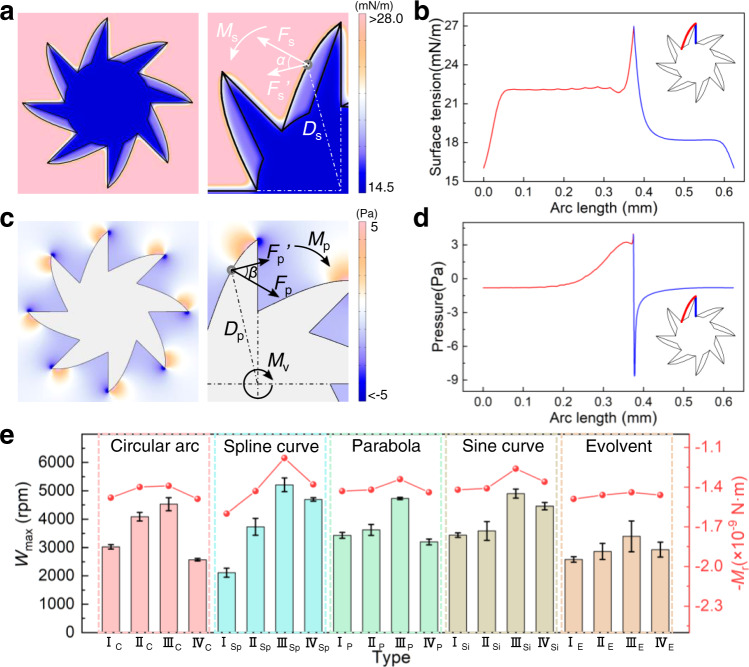
Surface tension distribution and shape design for drag reduction. **a** Simulation of surface tension distribution after hydrogel rotor is placed at the air-water interface for 43 ms. The enlarged view on the right shows the force *F*_s_ on a certain point on the rotor profile due to surface tension. The decomposed force *F*_s_′ provides a rotational torque on the rotor, and the sum of surface tension torques at all points along the rotor profile is denoted as *M*_s_. **b** Distribution curve of surface tension with the arc length on one tooth of hydrogel rotor. **c** Simulation of pressure distribution around hydrogel rotor. The enlarged view on the right shows the force *F*_p_ on a certain point on the rotor profile due to the pressure of water. The decomposed force *F*_p_′ provides a pressure resistance torque on the rotor, and the sum of pressure resistance torques at all points along the rotor profile is denoted as *M*_p_. The bottom surface of hydrogel rotor is subjected to viscous resistance torque, which is denoted as *M*_v_. **d** Distribution curve of water pressure with arc length on one tooth of hydrogel rotor. **e** The actual maximum rotation speed *W*_max_ and the theoretical resultant resistance torque *M*_r_ under different rotor shapes. In order to compare the influence of rotor shape on drag reduction, the resultant resistance torque *M*_r_ is calculated under the same rotation speed. Error bars denote the standard deviation of the measurements.

First of all, the surface tension distribution around the rotor is related to time, initial fuel concentration, diffusion coefficient and rotor shape. Through experiments (Supplementary Fig. 10), we found that the rotor can reach maximum rotation speed within 43 ± 3 ms after being placed at the air-water interface. Therefore, 43 ms was selected as the moment for simulating the surface tension distribution. Due to the surface porosity of different parts of the rotor is different (Fig. 1c), initial fuel concentration and diffusion coefficient of different parts of the rotor are also different. The initial HFIP fuel concentration is directly determined by the surface porosity. According to the model detailed in Supplementary Note 2, the HFIP diffusion coefficients of the low-porosity parts and the high-porosity parts are 1.47 × 10^−9^ m^2^/s and 2.25 × 10^−9^ m^2^/s, respectively. To analyze the surface tension distribution, we modeled the HFIP release as diffusion from a fixed source. The distribution of surface tension around the asymmetric rotor (“asymmetric” means that the surface porosity of each part of the rotor is different) is determined by simulation after the rotor is placed at the air–water interface for 43 ms (Fig. 2a). To be more intuitive, we plotted the distribution curve of surface tension with the arc length on one tooth of the asymmetric rotor (Fig. 2b).

As shown in the enlarged view in Fig. 2a, we established a two-dimensional dynamic model to analyze the influence of surface tension on the rotor. When the rotor is placed at the air-water interface, a point on the rotor profile is subjected to a force *F*_s_ due to surface tension. Typically, force *F*_s_ can be obtained by

1 $${F}_{s}=\gamma \cdot {dl}$$

where *γ* is the in-plane component of the surface tension at this point and *dl* is length of differential element. The force *F*_s_ is always perpendicular to the edge of the rotor. The decomposed force *F*_s_′ (perpendicular to the straight line connected to the rotor center) contributes to a surface tension torque to the rotor. The sum of surface tension torques at all points along the rotor profile can be calculated as follows

2 $${M}_{s}={\oint }_{L}\gamma \cdot {D}_{s}\cdot \,\cos \alpha \cdot dl$$

where *L* is the profile of rotor, *D*_s_ is the length from the differential element to the rotor center, *α* is the angle between component force *F*_s_′ and force *F*_s_. Based on the simulation results, the surface tension torque *M*_s_ of asymmetric rotor at 43 ms is 1.62 × 10^−9^ N m through numerical integration. For comparison, we also simulated the distribution of surface tension around a symmetric rotor with the same shape as the asymmetric rotor (“symmetric” means that the surface porosity of each part of the rotor is the same) and plotted the distribution curve of surface tension with the arc length on one tooth (Supplementary Fig. 11). According to the simulation results and numerical integration, the surface tension torque *M*_s_ of symmetric rotor is only 0.99 × 10^−9^ N m. Hence, by introducing asymmetric porosity into the rotor, the surface tension torque *M*_s_ is increased by ~64%. For the influence of rotor shape on the surface tension torque *M*_s_, we applied 20 common curves (5 types, including circular arc, spline curve, parabola, sine curve, and evolvent) to the design of rotor shape and theoretically calculated the surface tension torque *M*_s_ under different rotor shapes (Supplementary Table 2). The surface tension torques of asymmetric rotors with different shapes are all around 1.62 × 10^−9^ N m (min. 1.58 × 10^−9^ N m, max. 1.68 × 10^−9^ N m). To sum up, the influence of rotor shape on the surface tension torque is almost negligible under the premise that the rotor has asymmetric porosity, while the asymmetric porosity plays a leading role in enhancement of surface tension torque *M*_s_ which can improve the performance of hydrogel rotor.

Next, we further improved the performance of the hydrogel rotor through drag reduction. According to the general method described in Supplementary Note 3, the effect of drag reduction is judged by theoretically calculating the resultant resistance torque of the rotor with different shapes at the same rotation speed. When the rotor rotates at the interface of water and air, it is mainly subjected to two resistance torques (viscous resistance torque *M*_v_ and pressure resistance torque *M*_p_). The viscous resistance torque *M*_v_ of the rotor is evaluated by

3 $${M}_{v}=4.928\pi \eta \rho {r}^{4}{\omega }^{1.5}{\nu }^{0.5}$$

where *η* is the coefficient of the rotor shape, *ρ* is the density of the water, *ω* is the angular velocity of the rotor, *ν* is the kinematic viscosity of the water. As for pressure resistance torque, it is caused by the pressure of water on the submerged side wall of rotor during the rotation. A point on the rotor profile is subjected to a force *F*_p_ (Fig. 2c), which can be evaluated by

4 $${F}_{p}=P\cdot h\cdot {dl}$$

where *P* is pressure of water at this point, *h* is the depth to which the rotor is immersed in water (approximately equal to the thickness of the rotor), *dl* is length of differential element. The force *F*_p_ is always perpendicular to the edge of the rotor. The decomposed force *F*_p_′ (perpendicular to the straight line connected to the rotor center) contributes to a pressure resistance torque to the rotor. The sum of pressure resistance torques can be calculated as follows

5 $${M}_{p}={\oint }_{L}P\cdot h\cdot {D}_{p}\cdot \,\cos \beta \cdot dl$$

where *L* is the profile of rotor, *D*_p_ is the length from the differential element to the rotor center, *β* is the angle between component force *F*_p_′ and force *F*_p_.

The design of rotor shape for drag reduction is to minimize the resultant resistance torque *M*_r_ (sum of *M*_v_ and *M*_p_). As shown in Supplementary Table 3, we applied 20 common curves (5 types, including circular arc, spline curve, parabola, sine curve and evolvent) to the design of rotor shape, and studied the resistance torques on the rotor under different curves. Among the 20 types, the rotor with shape Type-III_Sp_ has the smallest resultant resistance torque *M*_r_ under the same rotation speed (line graph in Fig. 2e). For comparison, we also measured the actual maximum rotation speed *W*_max_ under these 20 types (histogram in Fig. 2e). When Type-III_Sp_ is adopted as the rotor shape, the *W*_max_ is the highest, reaching 5215 ± 240 rpm. The simulation result is consistent with the experiment, and Type-III_Sp_ is adopted as the optimized drag reduction shape for the rotor.

The distribution of water pressure around the Type-III_Sp_ rotor (Fig. 2c) is determined by simulation when the rotor reaches the actual maximum rotation speed (5215 rpm). To be more intuitive, we plotted the distribution curve of the pressure with the arc length on one tooth of the rotor (Fig. 2d). Based on the simulation results and theoretical calculations, resultant resistance torque *M*_r_ of the rotor (*r* = 250 µm, *h* = 474 µm and *n* = 8, Type-III_Sp_) at 5215 rpm is 1.57 × 10^−9^ N m, which is in agreement with the theoretical surface tension torque *M*_s_ (1.62 × 10^−9^ N m).

### High-performance Marangoni hydrogel rotors

**Fig. 3:**
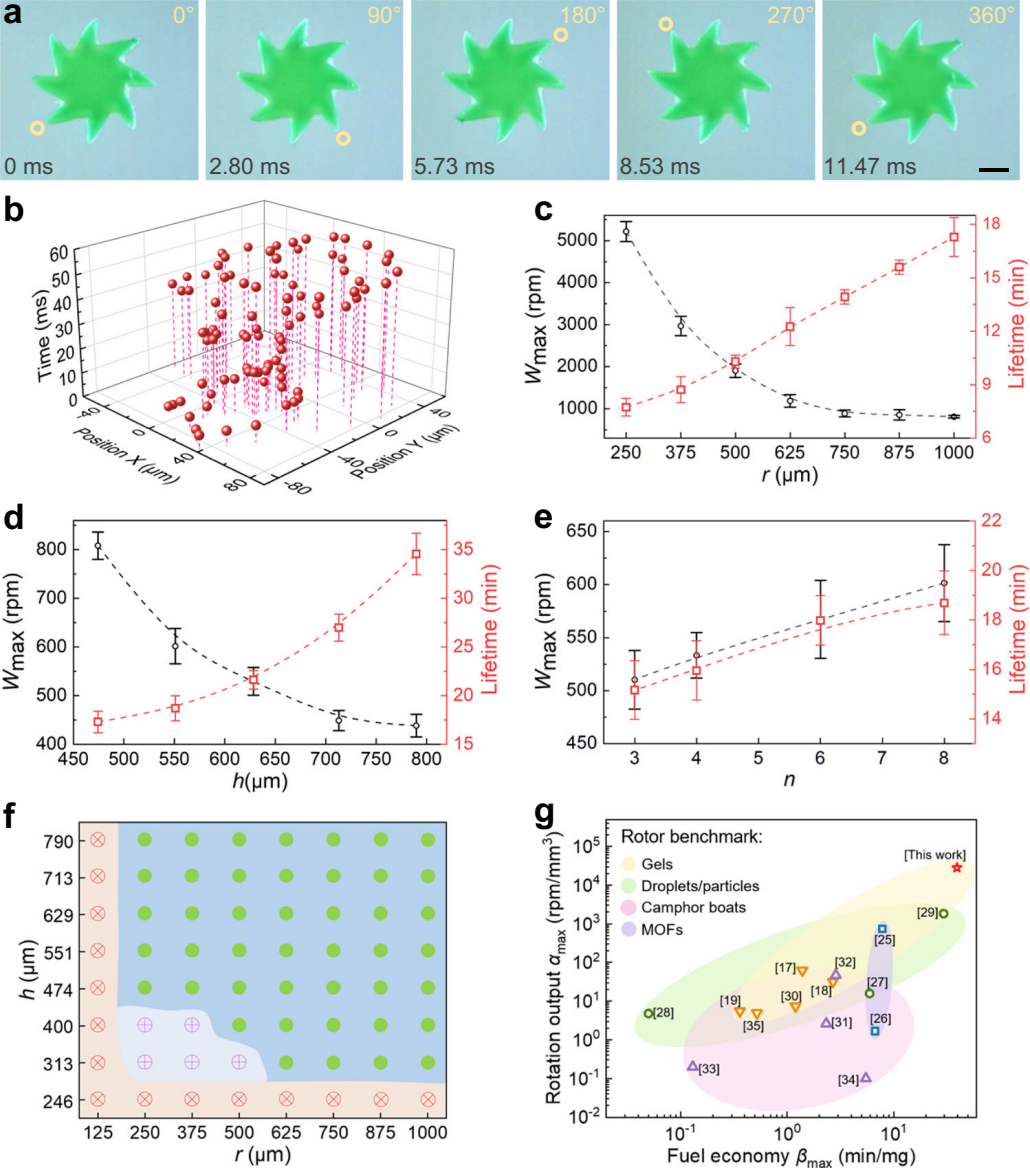
Performance of Marangoni hydrogel rotors. **a** High-speed camera images of a characteristic rotor (*r* = 250 µm, *h* = 474 µm and *n* = 8) during its rotation. Small yellowish circles are used to mark the rotation position of the rotor. **b** The motion track of the hydrogel rotor within 60 ms. **c**–**e** The relationships between the maximum rotation speed *W*_max_, lifetime and geometric parameters of hydrogel rotor. The thickness *h* and teeth number *n* remain unchanged at 474 µm and 8 in (**c**), respectively. The radius *r* and teeth number *n* remain unchanged at 1000 µm and 8 in (**d**), respectively. The radius *r* and thickness *h* remain unchanged at 1000 µm and 551 µm in (**e**), respectively. Black circles are *W*_max_, red squares are lifetime. Error bars denote the standard deviation of the measurements. **f** The phase diagram revealing rotational stability of hydrogel rotor with different geometric parameters. The green dots represent that the rotors can rotate stably in situ. Pink cross symbols indicate that the rotors can rotate but not in situ. The red cross symbols indicate that the rotors cannot rotate. **g** Chemical Marangoni rotor benchmark. Significant metrics for comparing performance of Marangoni rotors are rotation output *α*_max_ (maximum rotation speed per unit volume) and fuel economy *β*_max_ (maximum lifetime per unit fuel). Bio-inspired hydrogel rotors in this work outperform other chemical Marangoni rotors from a diversity of materials and fuels. Yellow inverted triangles are “Gels”, green circles are “Droplets/particles”, purple triangles are “Camphor boats”, blue squares are “MOFs”. Hydrogel rotors in this work belong to the category of “Gels”. Detailed values are given in Supplementary Table 4. The scale bar is 250 µm in (**a**).

Performance of Marangoni hydrogel rotors was analyzed using deionized (DI) water as the swimming medium. The rotors accelerated as they came in contact with water surface, instantly reaching a maximum rotation speed *W*_max_ and slowly decreasing rotation speed during their lifetime. Figure 3a shows the high-speed camera images of a characteristic rotor (*r* = 250 µm, *h* = 474 µm, and *n* = 8) at maximum rotation speed (intercepted from Supplementary Movie 1). It only took 11.47 ms for the rotor to make one rotation. Due to the high precision of grayscale digital light processing, the hydrogel rotors have high shape accuracy. Therefore, the rotor exhibited high position accuracy during the rotation, and the position offset in the plane was less than 120 µm (Fig. 3b). When other geometric parameters remain unchanged, the maximum rotation speed *W*_max_ decreases with the increase of radius *r* and thickness *h*, while *W*_max_ increases with the increase of teeth number *n*, respectively (Fig. 3c–e). This is mainly determined by the competitive relationship between the resultant torque and rotational inertia due to changes of these three geometric parameters. Similarly, when other geometric parameters remain unchanged, the lifetime of the rotors increases with the increase of radius *r*, thickness *h* and teeth number *n*, respectively (Fig. 3c–e). This is mainly because these three geometric parameters change the volume of the rotors and affect the amount of fuel carried by the rotors. We also studied the effect of the ratio of rotor inner circle radius to tooth length on the maximum rotation speed and lifetime (Supplementary Fig. 12). The rotor reached peak velocity up to 5215 ± 240 rpm with the specific geometrical parameters (*r* = 250 µm, *h* = 474 µm and *n* = 8) (Fig. 3c and Supplementary Movie 1) and peak lifetime up to 34.6 ± 2.1 min with the specific geometrical parameters (*r* = 1000 µm, *h* = 790 µm, and *n* = 8) (Fig. 3d, Supplementary Movie 2). In addition, the radius *r* and thickness *h* have the decisive influence on rotation stability of the rotors, while the teeth number *n* has nearly no effect on it. Figure 3f shows the rotation stability of the rotors under different combinations of radius *r* and thickness *h*. The green dots represent that the rotors can rotate stably in situ, the pink cross symbols indicate that the rotors can rotate but not in situ and the red cross symbols indicate that the rotors cannot rotate.

Since the rotor only carries limited fuel, the rotation output and fuel economy are used to evaluate the performance of our bio-inspired hydrogel rotor and compared with previous reports on analogous rotors based on Marangoni effect. The benchmark of chemical Marangoni rotors uses metrics, including rotation output *α*_max_ = *W*_max_/*V*_rotor_ (maximum rotation speed per unit volume of rotor) and fuel economy *β*_max_ = *t*_lifetime_/*m*_fuel_ (maximum lifetime per unit mass of fuel)^25,36^. Under these standards, our bio-inspired hydrogel rotors have obvious advantages over other chemical Marangoni rotors, including gels, droplets/particles, camphor boats and MOFs (Fig. 3g, Supplementary Table 4). The rotation output *α*_max_ of our hydrogel rotor is about 15 times the best work that has been reported, and the fuel economy *β*_max_ is ~34% higher than the best work that has been reported. Similarly to the reported by Pena-Francesch et al.^36^, the significant improvement in rotation output and fuel economy can be mainly attributed to the following factors: (a) design and fabrication of the rotor (shape design for reducing drag forces, asymmetric porosity for enhancing surface tension torque), (b) large Marangoni propulsive forces provided by little amount of fuel due to extremely low surface tension of HFIP fuel and (c) long lifetime caused by slow release of the HFIP fuel (using hydrogel chains to trap HFIP molecules).

### Rapid refueling of Marangoni hydrogel rotors

**Fig. 4:**
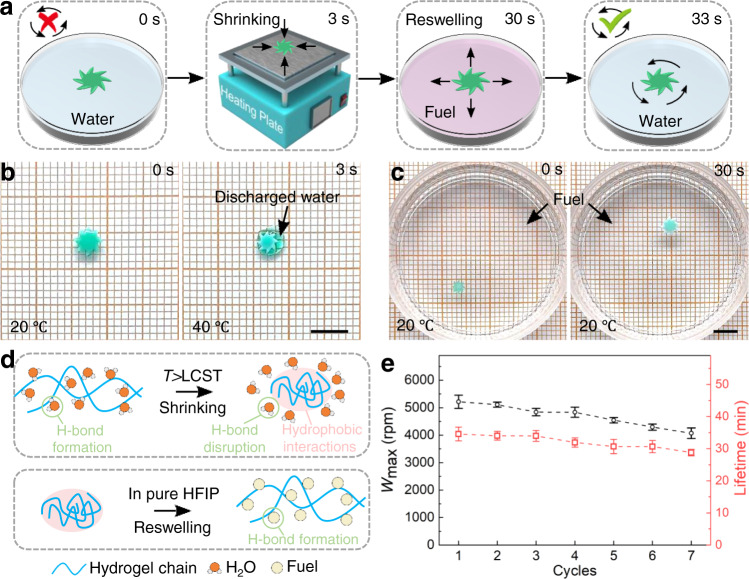
Rapid refueling of Marangoni hydrogel rotors. **a** Schematic diagram of refueling process. The whole refueling process of hydrogel rotor takes 33 s: shrinking and discharging water for 3 s, reswelling and absorbing HFIP fuel for 30 s. **b** Images of shrinking and discharging water process of hydrogel rotor. The temperature of heating plate is set to 40 °C. **c** Images of reswelling and absorbing HFIP fuel process of hydrogel rotor. **d** The mechanism of water removal and fuel re-injection. **e** Changes of maximum rotation speed *W*_max_ and lifetime of hydrogel rotors under multiple refueling cycles. The geometric parameters of hydrogel rotors for cyclic test of maximum rotation speed: *r* = 250 µm, *h* = 474 µm, and *n* = 8. The geometric parameters of hydrogel rotors for cyclic test of lifetime: *r* = 1000 µm, *h* = 790 µm, and *n* = 8. Error bars denote the standard deviation of the measurements. The scale bars are 5 mm in (**b** and **c**).

As the rotor rotates at the air-water interface, the fuel trapped in the rotor will eventually be exhausted and the rotor will stop rotating, which is an intrinsic limitation. One way to make the rotor recover rotation is refueling, while the refueling of most reported Marangoni rotors (e.g., droplet/particle rotors and MOF rotors) is infeasible or cumbersome^26–29^. Our hydrogel rotor can be refueled and the refueling process is divided into two steps: the first is the removal of water in the rotor (the original fuel molecules in hydrogel chains have been replaced by water molecules), and the second is the re-injection of fuel. Choosing PNIPAm hydrogel as the main frame of our rotor makes these two steps extremely simple and rapid (Fig. 4a). PNIPAm has lower critical solution temperature (LCST) behavior at ~32 °C in water^42–44^. When the temperature (e.g., 40 °C) is above its LCST, hydrogen bonds (H-bonds, between water molecules and amide segments in PNIPAm) are weakened. Thus, hydrophobic interactions between the hydrophobic backbone and isopropyl segments become dominant and cause the polymer chains inside PNIPAm to deform and shrink (Fig. 4d)^42^. As a result, the hydrogel rotor shrinks considerably and discharges water from its inside (Fig. 4b). When the shrinking hydrogel rotor is placed into pure HFIP, the amide segments in PNIPAm form H-bonds with HFIP molecules, which leads to the reswelling of the hydrogel rotor (Fig. 4c, d).

Due to the rapid LCST behavior of PNIPAm hydrogel, the step of water discharging only took 3 s (Fig. 4b, Supplementary Movie 3). And the step of HFIP fuel re-injection cost 30 s (Fig. 4c and Supplementary Movie 4). In this way, after the rotor stopped rotating because of the fuel exhaustion, it only took 33 s to make the rotor recover rotation. In addition, our rotors showed great stability and repeatability under multiple refueling cycles (Fig. 4e). The slight decreases in rotor performance (maximum rotation speed and lifetime) may be caused by the fatigue of the hydrogel^45,46^.

### Marangoni hydrogel rotors for kinetic energy transmission and mini-generator

**Fig. 5:**
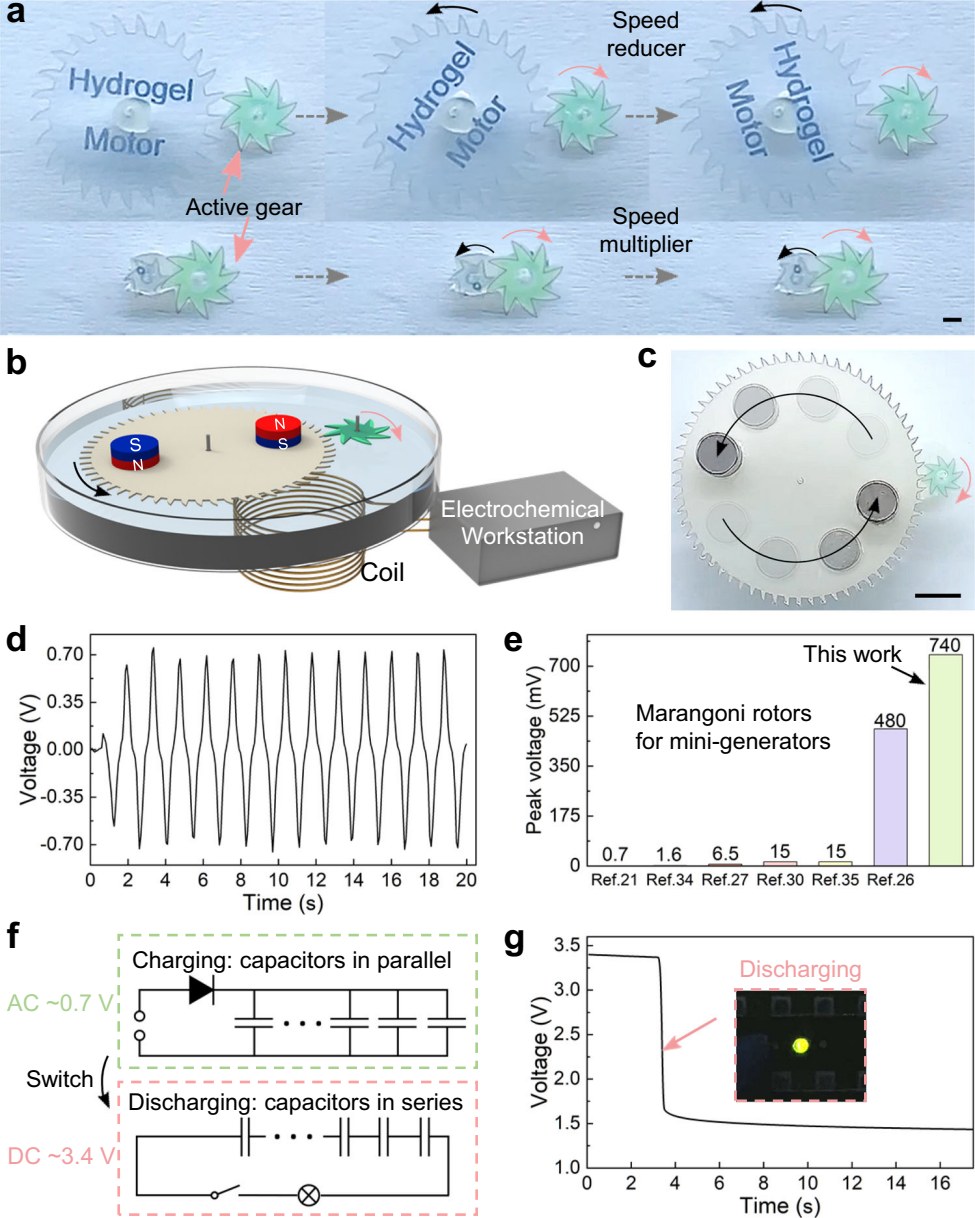
Marangoni hydrogel rotors for kinetic energy transmission and mini-generator. **a** Autonomous gear trains (speed reducer and multiplier) as motors. **b** Illustration of the mini-generator. The copper wire coil is connected to an electrochemical workstation to measure the induced voltage. **c** Time-lapse image of hydrogel rotor driving passive rotor with two magnets. **d** Induced voltage generated by the mini-generator with time. **e** Comparison of induced peak voltage in this work and that in previously reported works. **f** A high voltage can be obtained by half-wave rectification and switching the connection mode of ten charged capacitors. **g** Voltage-time curve of the capacitors when discharging to light an LED bulb (inset image). The scale bars are 1 mm in (**a**), 5 mm in (**c**).

Despite a lot of reports on chemical Marangoni rotors, most of them are only independent components that store organic fuel to realize rotation, and few of them realize functional applications^16–19,30–33,36^. It still remains a challenge to efficiently output the kinetic energy of chemical Marangoni rotors or convert it into other energy. For example, some rotors are very thin (e.g., tens of microns) in order to achieve higher rotation speed, which loses the possibility of outputting kinetic energy or converting it into other energy^16,25^. Besides, many rotors are constructed like “camphor boats” to utilize kinetic energy, which reduces the rotation output *α*_max_ and fuel economy *β*_max_^30–35^. Since our bio-inspired hydrogel rotors have large thicknesses (ranging from 246 µm to 790 µm) and superior rotation output *α*_max_ and fuel economy *β*_max_, they can directly supply energy to mechanical system through gear meshing (Fig. 5a). Inspired by the gear trains demonstration by Pena-Francesch et al.^36^, we also show kinetic energy transmission of our rotors as gear reducer/multiplier for over 20 min (Supplementary Movie 5, 6). The whole hydrogel rotor-based devices can be regarded as different motors, which can output required rotation speed and torque.

As a proof of concept, we also demonstrated the use of Marangoni hydrogel rotors for mini-generators (Fig. 5b). For the device assemble of the mini-generator, two capillary glass tubes were used to hold the hydrogel rotor and the passive rotor to keep them engaged. The passive rotor was integrated with two magnets, and the induced voltage was generated when there was relative motion between the magnets and the solenoid coil^21,26^. The size of the passive rotor and magnets, the number of magnets, and the distance between the magnets were optimized through many experiments. Since the induced voltage depends on the rotation speed of the passive rotor, we have reduced the resistance between the passive rotor and water through superhydrophobic treatment (Supplementary Fig. 13) to enable its movement more efficient^26^. Due to the superior rotation output *α*_max_ and fuel economy *β*_max_ of the hydrogel rotor, combined with the optimization of the structure and parameters of the passive rotor, the hydrogel rotor can drive the passive rotor to rotate at high speed for more than 8 min (Fig. 5c and Supplementary Movie 7). Supplementary Fig. 14 shows the change of induced voltage generated by the mini-generator in 500 s. According to Lenz’s law, the value of induced voltage is related to the change rate of magnetic flux^26,30,35^, so the induced voltage contains positive peaks (when the magnet is away from the coil) and negative peaks (when the magnet is close to the coil) (Fig. 5d). More remarkably, the maximum peak voltage can reach 740 mV, which is higher than those reported mini-generators based on Marangoni rotors (Fig. 5e)^21,26,27,30,34,35^.

For further practical application, we examined the possibility of the bio-inspired hydrogel rotor-based mini-generator to power other electronics. A circuit was designed to rectify the induced alternating current (AC) into the direct current (DC) and collect the electric energy in 10 capacitors connected in parallel (Supplementary Fig. 15)^47^. After charged by the mini-generator for 12 s, the open-circuit voltage of the 10 capacitors reached ~0.34 V. Then, the connection mode of the capacitors was switched to a serial manner, so that the open circuit voltage reached ~3.4 V (Fig. 5f), which was sufficient to directly power a light-emitting diode (LED) bulb (Fig. 5g and Supplementary Movie 8).

### Individual-specific locomotion of hydrogel rotor group under the same magnetic field

**Fig. 6:**
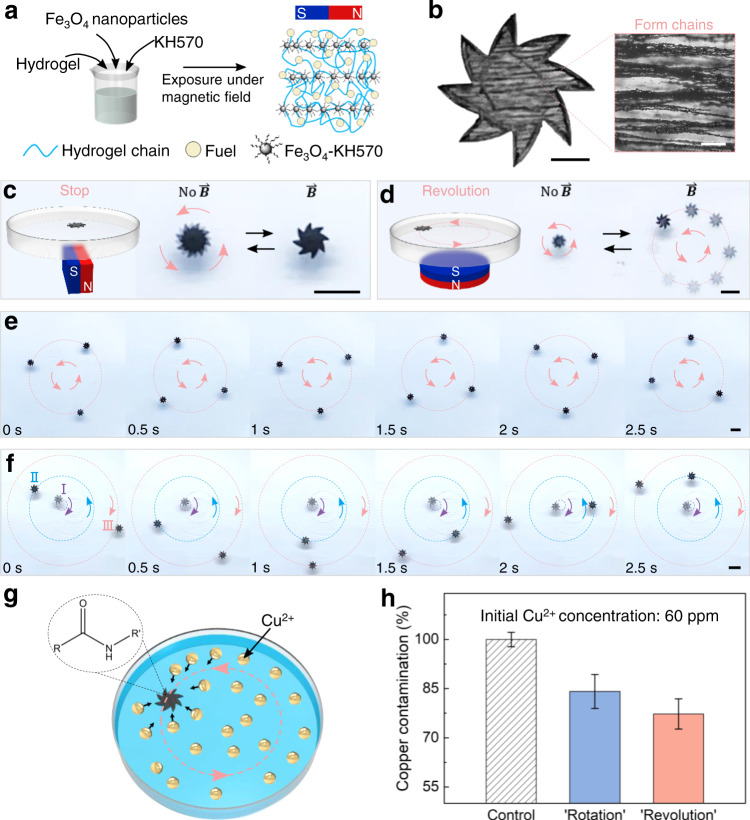
Controllable magnetic hydrogel rotors for environmental remediation. **a** Schematic illustration of the fabrication procedure of the magnetic hydrogel. Before UV exposure, the KH570-modified Fe_3_O_4_ nanoparticles distributed in the hydrogel precursor are magnetized to assemble into chains. **b** Optical microscopy images of magnetic hydrogel rotor and Fe_3_O_4_ nanoparticles chains. **c** By removing or applying the magnetic field of a cuboid magnet, the magnetic rotor can be switched between rotation and stop. **d** By removing or applying the magnetic field of a cylindrical magnet, the magnetic rotor can be switched between rotation and revolution. **e** Three magnetic hydrogel rotors with the same concentration of Fe_3_O_4_ nanoparticles can revolve along the same orbit under the magnetic field of a cylindrical magnet. **f** Three magnetic hydrogel rotors with different concentrations of Fe_3_O_4_ nanoparticles (15 mg/mL in I, 40 mg/mL in II, and 65 mg/mL in III) can revolve along different orbits under the magnetic field of a cylindrical magnet. The revolution direction of the rotor can be switched by turning the rotor over. **g** Scheme of water purification. Magnetic hydrogel rotors contain a large number of amide groups, which can combine with copper ions. **h** Under the same conditions, the efficiency of capturing Cu^2+^ contaminants from water by magnetic hydrogel rotors revolving under magnetic field is higher than that by the rotors rotating in situ. Error bars denote the standard deviation of the measurements. The scale bars are 1 mm in **b** (left), 100 µm in **b** (right), 5 mm in (**c**–**f**).

Locomotion control of most self-propelled systems is challenging due to their lack of directionality, resulting in a single propelling mode (for chemical Marangoni rotors, just “Rotation”). Here, we demonstrated both of “Rotation” and “Revolution” by magnetic steering. We fabricated magnetic hydrogel rotors by integrating superparamagnetic Fe_3_O_4_ nanoparticles into the hydrogel chains (Fig. 6a). The KH570-modified Fe_3_O_4_ nanoparticles were uniformly distributed in the hydrogel precursor by ultrasound. Under the magnetization of a horizontal magnetic field, Fe_3_O_4_ nanoparticles assembled into chains along the magnetic field direction (Supplementary Fig. 16). After DMD exposure, the Fe_3_O_4_ nanoparticle chains were fixed in the hydrogel rotor (Fig. 6b). Due to the strong magnetic response, the fixed Fe_3_O_4_ nanoparticle chains inside the rotor tend to be consistent with the direction of magnetic induction line, which brings magnetic responsiveness to the rotor. Under the magnetic field of a cuboid magnet, the magnetic rotor that originally rotated in situ would stop rotating (mechanical analysis is shown in Supplementary Fig. 17). When the magnetic field was removed, the rotor returned to rotate in situ. The switching of motion mode was continuously reversible (Fig. 6c and Supplementary Movie 9). Similarly, under the magnetic field of a cylindrical magnet, the magnetic rotor that originally rotated in situ would revolve (mechanical analysis is shown in Supplementary Fig. 18). When the magnetic field was removed, the rotor returned to rotate in situ. This motion mode switching was also continuously reversible (Fig. 6d and Supplementary Movie 10).

In order to demonstrate the accuracy and flexibility of motion control, we have achieved group control of magnetic hydrogel rotors and further individual-specific locomotion in group. Three magnetic rotors with the same Fe_3_O_4_ nanoparticles concentration could revolve along the same orbit (Fig. 6e and Supplementary Movie 11). What’s more, as shown in Fig. 6f and Supplementary Movie 12, three individual rotors with different Fe_3_O_4_ nanoparticles concentrations (details in Supplementary Fig. 19) could revolve along orbits of different radii due to the difference in magnetic responsiveness (the lower the concentration, the more inside). In addition, by turning the middle rotor over, the innermost and outermost rotors revolved clockwise, while the middle rotor revolved counterclockwise.

Although there have been reports of programmable locomotion of Marangoni swimmers, their motion forms are all “Translation” and they cannot achieve group control as well as further individual-specific locomotion in group^20,36^. Also, the direction of magnet needs to be constantly adjusted with the swimmer’s movement^36^. In contrast, our work focuses on “Revolution” and realizes the group control of hydrogel rotors as well as further individual-specific locomotion of hydrogel rotor group. The locomotion of individual rotor (e.g., direction and radius of revolution) can be accurately tuned when multiple rotors are controlled in group by the same magnetic field. Furthermore, the magnet does not need to move with the hydrogel rotor in the programmable locomotion, but only needs to be placed under the rotor and remain stationary.

To explore additional functionalities, we demonstrated the use of magnetic hydrogel rotors for water purification (Fig. 6g). Heavy metal water pollution is an increasing global concern and various techniques have been used to remove heavy metal ions (including copper ion) in water. Among them, hydrogel adsorption has been considered as a promising platform because of its high efficiency, low cost and easy handling^48,49^. In case of our magnetic hydrogel rotors, we take advantage of PNIPAm, which is the main frame of the rotors. PNIPAm is porous and contains a lot of amide groups, which are well-known chelators that bind to copper ions or other heavy metal ions^50–53^. Moreover, magnetic hydrogel rotors revolving under magnetic field are more efficient to capture Cu^2+^ contaminants from water than the rotors rotating in situ (Fig. 6h), because the revolving rotors have a larger motion area than the rotating rotors. Although the water purification efficiency can be further improved (e.g., using more rotors simultaneously), the results prove the heavy metal ion capturing capability of our bio-inspired hydrogel rotors for potential water purification applications, and add versatility to our self-propelled, controllable, and environmentally friendly platform.

## Discussion

In summary, inspired by rove beetle in genus *Stenus*, we have developed a self-propelled Marangoni rotor featuring with high performance, reusability, and programmable locomotion by employing PNIPAm as the main frame and anesthetic metabolite as the fuel. The low surface tension fuel (especially maintaining low surface tension at very low concentrations) can generate large surface tension gradients, which further form strong Marangoni forces to self-propel the rotor. Moreover, combined with asymmetric porosity for the further enhancement of surface tension torque and well-designed rotor profile for reducing drag, the hydrogel rotor presented in this work exceeds previous Marangoni rotors in terms of rotation output and fuel economy, making it the best comprehensive performance air–liquid interface rotor reported to date. Taking the advantage of high precision of grayscale digital light processing, our hydrogel rotor also exhibits high rotation position accuracy. The rapid refueling achieved by the thermal sensitivity of PNIPAm enables the reusability of the rotor. Iron-powder dopant further imparts the rotors with individual-specific locomotion in group under the magnetic stimuli. Furthermore, we demonstrate diverse functionalities of the hydrogel rotor, including kinetic energy transmission as speed reducer and multiplier, energy conversion as the mini-generator, and removal of heavy metal ions in water for environmental remediation. This work is envisioned to provide profound guidance for designing miniaturized rotating machineries in the fields of robotics, energy, and environment. Future work will explore the bio-inspired hydrogel rotors as the carriers and propulsion sources in natural or engineered air-liquid interfaces (e.g., physiologically relevant environments and interfacial chemical reactions) for sensing and targeted sustained releasing of substances (e.g., drugs and chemical reagents).

## Methods

### Preparation of thermoresponsive hydrogel precursor solution

N-Isopropylacrylamide, N,N’-Methylenebisacrylamide, dimethyl sulfoxide, deionized water, and 2-hydroxy-2-methylpropiophenone were mixed at a weight ratio of 10.9:0.6:15:5:1, followed by stirring for 12 h at 45 °C. The precursor was kept in yellow light condition to avoid unnecessary light exposure.

### Preparation of KH570-modified Fe_3_O_4_ nanoparticles

First, 6.5 g Fe_3_O_4_ nanoparticles (800 nm in diameter), 8 mL KH-570, 200 mL deionized water, and 200 mL ethanol were mixed by ultrasound for 5 min, followed by stirring for 5 h at 50 °C. Then, the suspension was left for 24 h and filtered. The resulting KH570-modified Fe_3_O_4_ powder was vacuum dried at 35 °C.

### Grayscale digital light processing

90 µL hydrogel precursor was dropped on a cover glass (20 × 20 mm) and then irradiated for 5 s with grayscale UV laser (λ = 375 nm) generated by DMD exposure system (Supplementary Fig. 2) to form hydrogel rotor. The exposure intensity of low surface porosity parts and high surface porosity parts of asymmetric rotor is 24 mW/cm^2^ and 12 mW/cm^2^, respectively. Hydrogel rotors with different thicknesses were obtained by controlling the volume of hydrogel precursor (Supplementary Fig. 5). For every increase of 35 µL of hydrogel precursor, the exposure time increased by 2 s.

### Characterization

The sample was freeze-dried by a freeze dryer (FD-1B-80, BIOCOOL, China) overnight. The SEM images were obtained using a secondary electron SEM (EVO18, ZEISS, Germany). The rotation of the hydrogel rotor was captured by a high-speed camera (Phantom VEO 710 s, AMETEK, USA). The optical images were taken by a charge-coupled device (CCD) camera (MV-SUA31GC-T, MindVision, China).

### Power generation experiments

A coil with 20,000 turns of copper wire (copper wire diameter: 0.05 mm, coil diameter: 8 cm) was hung near the NdFeB permanent magnets (diameter: 5 mm, height: 5 mm, magnetic flux density: ~450 mT). Two ends of the coil were connected to an electrochemical workstation (CHI760, CH Instruments, USA) to form a closed loop for displaying the induced voltage.

### Environmental remediation experiments

Anhydrous copper sulfate is dissolved in deionized water to a concentration of 60 ppm Cu^2+^ as reference. Three magnetic hydrogel rotors (*r* = 1000 µm, *h* = 790 µm and *n* = 8) were placed in 5 ml 60 ppm solution and rotated for 30 min without applying a magnetic field. As a comparison, another three magnetic hydrogel rotors with the same parameters were placed in 5 ml 60 ppm solution and revolved for 30 min under the magnetic field of a cylindrical magnet. The Cu^2+^ concentrations of the solutions were measured by inductively coupled plasma-optical emission spectrometry (iCAP 7400, Thermo, USA).

## Supplementary information

Supplementary Information

Description of Additional Supplementary Files

Supplementary Movie 1

Supplementary Movie 2

Supplementary Movie 3

Supplementary Movie 4

Supplementary Movie 5

Supplementary Movie 6

Supplementary Movie 7

Supplementary Movie 8

Supplementary Movie 9

Supplementary Movie 10

Supplementary Movie 11

Supplementary Movie 12

## Data Availability

All data needed to evaluate the conclusions in the paper are present in the manuscript and Supplementary Information. The data are also available upon request from the corresponding author.

## Code availability

This study did not use any custom code or mathematical algorithm that is deemed central to the conclusions.
