# Author Correction: High-performance Marangoni hydrogel rotors with asymmetric porosity and drag reduction profile

**DOI:** 10.1038/s41467-023-37461-5

**Published:** 2023-04-06

**Authors:** Hao Wu, Yiyu Chen, Wenlong Xu, Chen Xin, Tao Wu, Wei Feng, Hao Yu, Chao Chen, Shaojun Jiang, Yachao Zhang, Xiaojie Wang, Minghui Duan, Cong Zhang, Shunli Liu, Dawei Wang, Yanlei Hu, Jiawen Li, Erqiang Li, HengAn Wu, Jiaru Chu, Dong Wu

**Affiliations:** 1 CAS Key Laboratory of Mechanical Behavior and Design of Materials, Key Laboratory of Precision Scientific Instrumentation of Anhui Higher Education Institutes, Department of Precision Machinery and Precision Instrumentation, University of Science and Technology of China, Hefei, 230027 China; 2 Key Laboratory of Testing Technology for Manufacturing Process of Ministry of Education, Southwest University of Science and Technology, Mianyang, 621010 China; 3 Department of Modern Mechanics, University of Science and Technology of China, Hefei, 230026 China; 4 Physical Intelligence Department, Max Planck Institute for Intelligent Systems, 70569 Stuttgart, Germany

**Keywords:** Actuators, Mechanical engineering, Gels and hydrogels, Electrical and electronic engineering, Fluid dynamics

Correction to: *Nature Communications* 10.1038/s41467-022-35186-5, published online 3 January 2023

The original version of this article contained some textual overlap with previous published work by Pena-Francesch, A., Giltinan, J., Sitti, M. Multifunctional and biodegradable self-propelled protein motors. *Nat. Commun*. 10, 3188 (2019), ref. ^36^, and omitted relevant references in parts of its discussion. The new version of the main article has been corrected to minimize textual overlap and provide the appropriate references throughout the main text. Excerpts of the corrected and respective original sentences are listed below. These textual corrections do not change the results and claims in the original version of this article.

Furthermore, the original version of this article omitted a reference to previous work in ‘Zhang, L. N., Cheng, M. J., Luo, H., Zhang, H. B., Ju, G. N., Liu, P., Zhou, Y. F., Shi, F. Mini‐generator based on reciprocating vertical motions driven by intracorporeal energy. *Adv. Healthcare Mater.* 8, 1900060 (2019)’. This has been added as ref. ^47^ at the end of the second sentence of the third paragraph of sub-section ‘Marangoni hydrogel rotors for kinetic energy transmission and mini-generator’: ‘A circuit was designed to rectify the induced alternating current (AC) into the direct current (DC) and collect the electric energy in 10 capacitors connected in parallel (Supplementary Fig. 15)^47^.’ All subsequent references were renumbered to 48–57 accordingly.

**Excerpts of original and corrected text:**

In sub-section entitled ‘Propulsion mechanism of bio-inspired Marangoni hydrogel rotor’ from the ‘Results’ section.

**Original:** “... In the presence of water, the trapped HFIP molecules are replaced by the absorbed water molecules (Fig. 1e). When the rotor is placed on the water surface, HFIP is slowly released from the rotor to form a surface tension gradient which generates driving torque and propels the rotor to rotate at high speed.”

**Corrected:** “... In an aqueous environment, the water molecules around the rotor can replace the HFIP molecules trapped in the hydrogel chains^16–19,36^ (Fig. 1e). When the rotor is placed at the air-water interface, it can slowly secrete HFIP to form a surface tension gradient around itself and generate driving torque to drive itself to rotate rapidly^21,22,25^.”

**Original:** “... Moreover, the capture of HFIP in the hydrogel chains and its slow release through the porous structure permit for high storage of the fuel. ...”

**Corrected:** “... Moreover, the porous structure of the hydrogel and the capture of HFIP by the hydrogel chains guarantee high storage and slow release of fuel^16–19^. ...”

**Original:** “... This rotor-fuel synergy in our PNIPAm hydrogel-based system, together with special design (enhancement of surface tension torque by asymmetric porosity and reduction of drag by well-designed shape), result in excellent performance of the rotors (i.e., high rotation speed and long lifetime). ...”

**Corrected:** “... Due to the synergy of hydrogel-fuel, enhancement of surface tension torque by asymmetric porosity and reduction of drag by well-designed shape, the rotor exhibits excellent performance including high rotation speed and long lifetime. ...”

**Original:** “... This expands the applicability of the bio-inspired hydrogel rotors and permits the utilization in biological and physiologically relevant environments. ...”

**Corrected:** “... This expands the applicability of the bio-inspired hydrogel rotors and permits the utilization in biological applications^36^. ...”

In sub-section entitled ‘High-performance Marangoni hydrogel rotors’ from the ‘Results’ section.

**Original:** “... We attribute such superior rotation output and fuel economy to the combination of: (a) design and fabrication of the rotor (shape design for reducing drag forces, asymmetric porosity for enhancing surface tension torque), (b) extremely low surface tension of HFIP fuel (large Marangoni propulsive forces with tiny fuel) and (c) capture of fuel in the hydrogel chains (slow release of the fuel and long lifetime of the rotor).”

**Corrected:** “... Similarly to the reported by Pena-Francesch et al.^36^, the significant improvement in rotation output and fuel economy can be mainly attributed to the following factors: (a) design and fabrication of the rotor (shape design for reducing drag forces, asymmetric porosity for enhancing surface tension torque), (b) large Marangoni propulsive forces provided by little amount of fuel due to extremely low surface tension of HFIP fuel and (c) long lifetime caused by slow release of the HFIP fuel (using hydrogel chains to trap HFIP molecules).”

In sub-section entitled ‘Marangoni hydrogel rotors for kinetic energy transmission and mini-generator’ from the ‘Results’ section.

**Original:** “Although various chemical Marangoni rotors have been reported to date, the majority of the rotors are non-functional passive elements which are used solely for fuel storage purposes^16–19,30^^–33^. ...”

**Corrected:** “Despite a lot of reports on chemical Marangoni rotors, most of them are only independent components that store organic fuel to realize rotation, and few of them realize functional applications^16–19,30–33,36^. ...”

**Original:** “... Since our bio-inspired hydrogel rotors have large thicknesses (ranging from 246 to 790 µm) and superior rotation output *α*_max_ and fuel economy *β*_max_, they can be applied to power autonomous mechanisms, such as gear trains (Fig. 5a). We demonstrated kinetic energy transmission of our rotors in speed reducer and multiplier gear trains for at least 20 min (Supplementary Movies 5, 6). ...”

**Corrected:** “... Since our bio-inspired hydrogel rotors have large thicknesses (ranging from 246 µm to 790 µm) and superior rotation output *α*_max_ and fuel economy *β*_max_, they can directly supply energy to mechanical system through gear meshing (Fig. 5a). Inspired by the gear trains demonstration by Pena-Francesch et al.^36^, we also show kinetic energy transmission of our rotors as gear reducer/multiplier for over 20 min (Supplementary Movie 5, 6). ...”

**Original:** “... The passive rotor was integrated with two magnets, and the electric potential was induced as the magnets pass under a solenoid coil. The size of the passive rotor and magnets, the number of magnets, and the distance between the magnets were optimized through many experiments. Because the intensity of peak voltage is proportional to the rotation speed of passive rotor, in order to make the motion of passive rotor more effective, the friction with the water interface is minimized by superhydrophobic treatment (Supplementary Fig. 13). ...”

**Corrected:** “... The passive rotor was integrated with two magnets, and the induced voltage was generated when there was relative motion between the magnets and the solenoid coil^21,26^. The size of the passive rotor and magnets, the number of magnets, and the distance between the magnets were optimized through many experiments. Since the induced voltage depends on the rotation speed of the passive rotor, we have reduced the resistance between the passive rotor and water through superhydrophobic treatment (Supplementary Fig. 13) to enable its movement more efficient^26^. ...”

**Original:** “... Since voltage is proportional to the change of magnetic flux per time by Lenz’s law, the induced voltage should consist of a pair of negative and positive peaks (Fig. 5d). The negative peak is generated when the magnet enters the coil area and the positive peak is induced as the magnet leaves the area. ...”

**Corrected:** “... According to Lenz’s law, the value of induced voltage is related to the change rate of magnetic flux^26,30,35^, so the induced voltage contains positive peaks (when the magnet is away from the coil) and negative peaks (when the magnet is close to the coil) (Fig. 5d). ...”

**Original:** “... To transfer the induced alternating current (AC) into the direct current (DC), we designed a circuit acting as a rectifier to collect electric energy in 10 capacitors connected in parallel (Supplementary Fig. 15). After charged by the mini-generator for 12 s, the open-circuit voltage of the 10 capacitors reached ~0.34 V. Then, the connection mode of the capacitors was switched to a serial manner, so that the open circuit voltage reached ~3.4 V (Fig. 5f), which was high enough to power electronics such as the light-emitting diode (LED) bulb (Fig. 5g and Supplementary Movie 8).”

**Corrected:** “... A circuit was designed to rectify the induced alternating current (AC) into the direct current (DC) and collect the electric energy in 10 capacitors connected in parallel (Supplementary Fig. 15)^47^. After charged by the mini-generator for 12 s, the open-circuit voltage of the 10 capacitors reached ~0.34 V. Then, the connection mode of the capacitors was switched to a serial manner, so that the open circuit voltage reached ~3.4 V (Fig. 5f), which was sufficient to directly power a light-emitting diode (LED) bulb (Fig. 5g and Supplementary Movie 8).”

These have been corrected in both the PDF and HTML versions of the article.

