# Supplementary material for: High-performance Marangoni hydrogel rotors with asymmetric porosity and drag reduction profile
Source: Nat Commun. 2023 Jan 3;14:20. doi: 10.1038/s41467-022-35186-5 (PMC9810638; doi:10.1038/s41467-022-35186-5)
Supplement: Supplementary file 1 — Supplementary Information [file 41467_2022_35186_MOESM1_ESM.pdf]

Supplementary Information

High-performance Marangoni hydrogel rotors with asymmetric porosity and drag reduction profile

*Hao Wu[1], Yiyu Chen[1,2], Wenlong Xu[3], Chen Xin[1], Tao Wu[3], Wei Feng[4], Hao Yu[3], Chao Chen[1], Shaojun Jiang[1], Yachao Zhang[1], Xiaojie Wang[1], Minghui Duan[1], Cong Zhang[1], Shunli Liu[1], Dawei Wang[1], Yanlei Hu[1], Jiawen Li[1], Erqiang Li[3], HengAn Wu[3], Jiaru Chu[1], and Dong Wu[1]**

[1]CAS Key Laboratory of Mechanical Behavior and Design of Materials, Key Laboratory of Precision Scientific Instrumentation of Anhui Higher Education Institutes, Department of Precision Machinery and Precision Instrumentation, University of Science and Technology of China, Hefei 230027, China.

[2]Key Laboratory of Testing Technology for Manufacturing Process of Ministry of Education, Southwest University of Science and Technology, Mianyang 621010, China.

[3]Department of Modern Mechanics, University of Science and Technology of China, Hefei 230026, China.

[4]Physical Intelligence Department, Max Planck Institute for Intelligent Systems, 70569 Stuttgart, Germany.

*Corresponding author. Email: dongwu@ustc.edu.cn (D.W.)

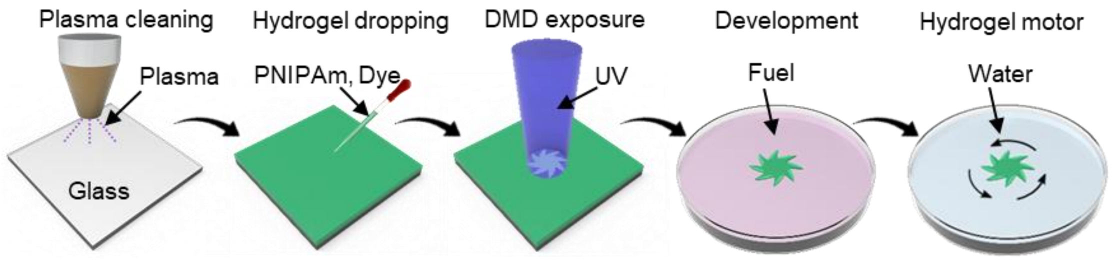

Supplementary Figure 1. Bio-inspired hydrogel rotor fabrication. First, a square cover glass (20×20 mm) was treated by plasma cleaning to obtain super-hydrophilicity. Then, the hydrogel (with green dyes added for enhanced visualization) was dropped on the cover glass. Since the cover glass was already super-hydrophilic and the solvents of the hydrogel were water and dimethyl sulfoxide (DMSO), the hydrogel was spread spontaneous and evenly on the cover glass to form a liquid film. The liquid film was exposed by a DMD (Digital Micromirror Device) exposure system to form specified rotor shape, and the rotor was then transferred into hexafluoroisopropanol (HFIP) for development and fuel filling.

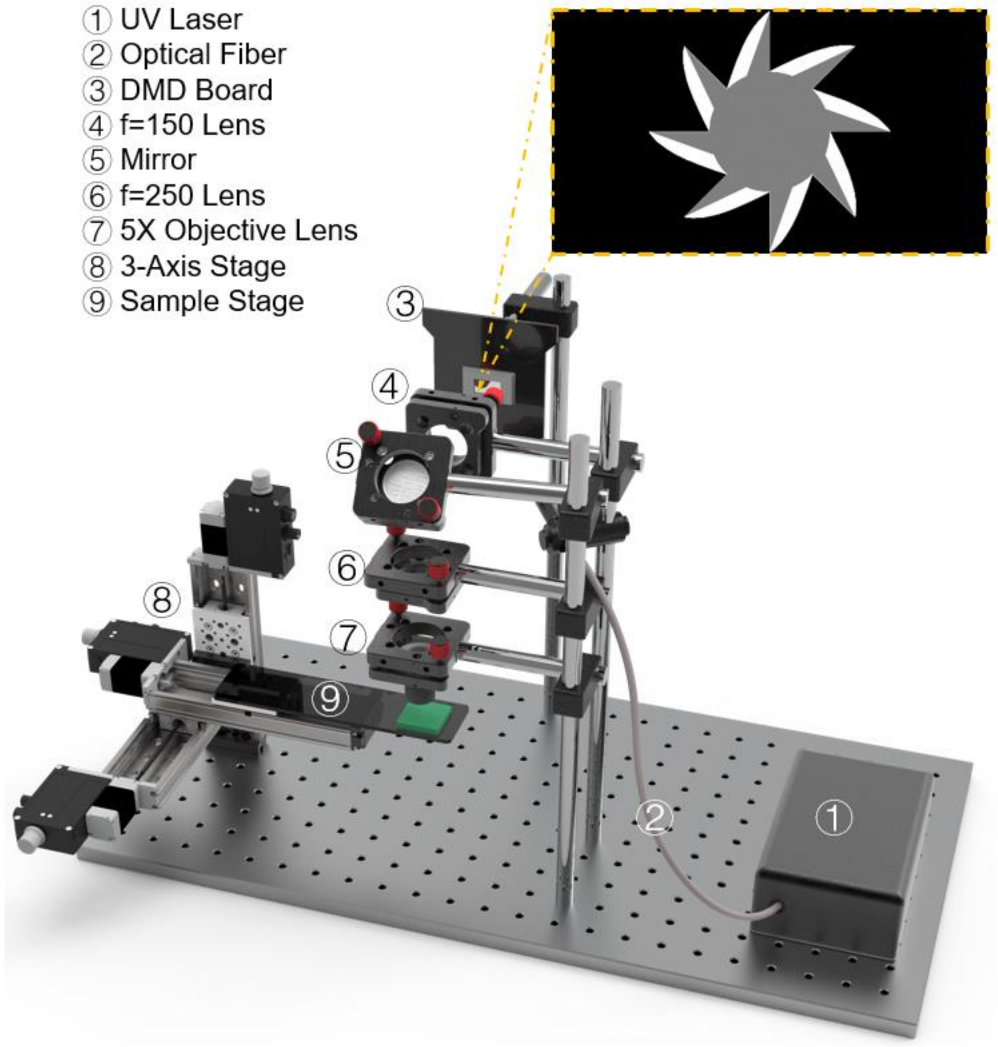

Supplementary Figure 2. The grayscale digital light processing based on DMD. Inset is the grayscale image used to process the asymmetric hydrogel rotor.

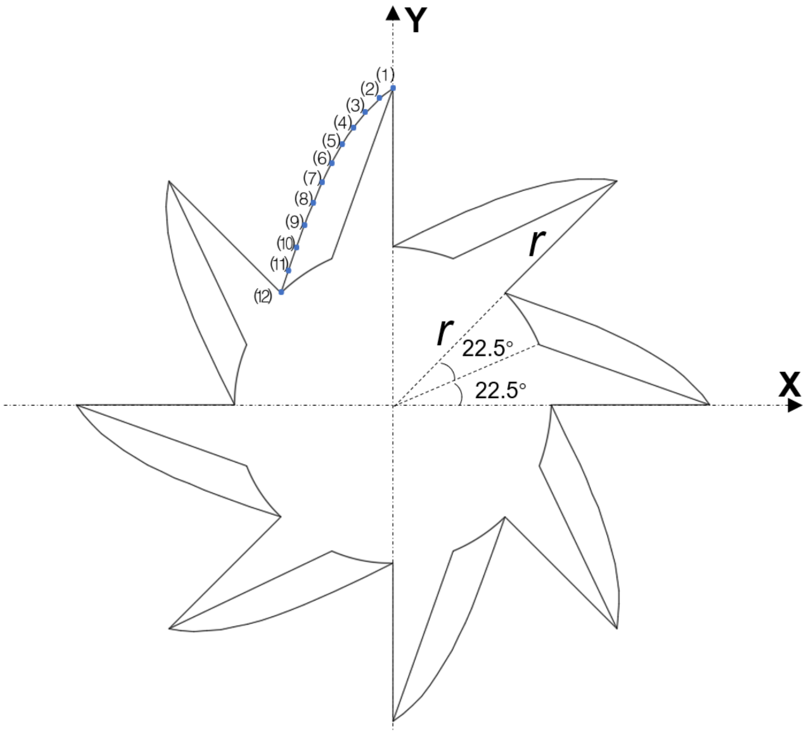

Supplementary Figure 3. After establishing the coordinate system with the center of the rotor, the coordinates of the 12 control points of the spline curve: (0, 0.5000), (-0.0229, 0.4821), (-0.0438, 0.4609), (-0.0630, 0.4368), (-0.0806, 0.4100), (-0.0969, 0.3810), (-0.1120, 0.3499), (-0.1261, 0.3173), (-0.1395, 0.2833), (-0.1522, 0.2483), (-0.1646, 0.2127), (-0.1768, 0.1768). The coordinates are in millimeters and *r* is 250 μm.

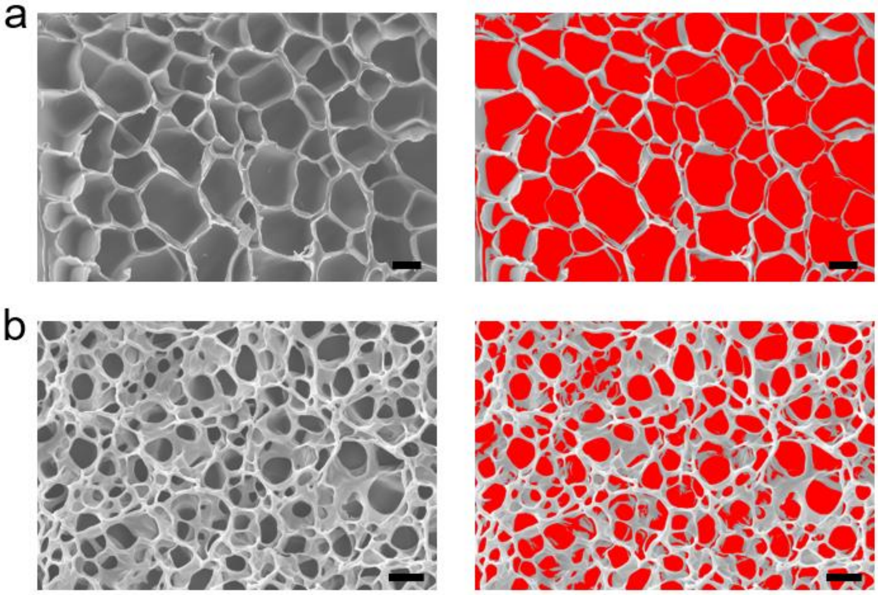

Supplementary Figure 4. Measurement of surface porosity of hydrogels using ImageJ analysis software (National Institutes of Health, Maryland, USA). (a) SEM images of the hydrogel with low crosslinking density (using low exposure dose) before and after image identification. (b) SEM images of the hydrogel with high crosslinking density (using high exposure dose) before and after image identification. Firstly, we reset the scale bar in setting and converted the image to 8-bit format. Then, we adjusted the threshold to select the pores by software. Finally, we measured the surface porosity in set measurement. Five SEM images of different regions were taken to measure the surface porosity. The surface porosity of the hydrogel with low crosslinking density is 78.8 ± 2.0% and the surface porosity of the hydrogel with high crosslinking density is 40.8 ± 1.7%. The scale bars are 20 μm in (a), 10 μm in (b).

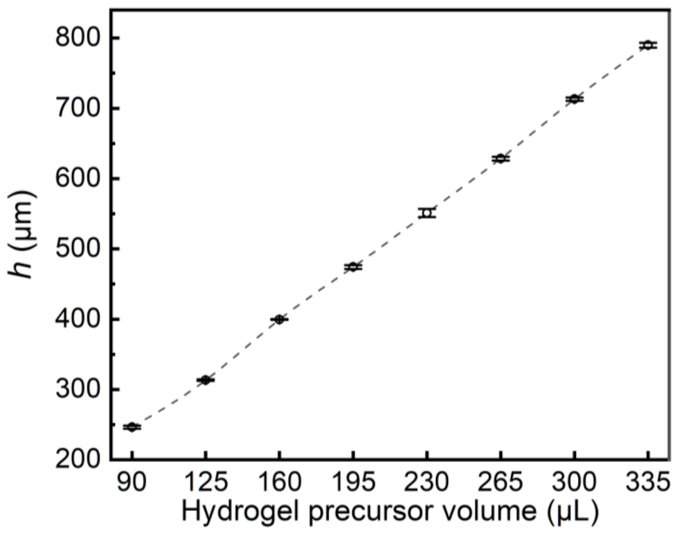

Supplementary Figure 5. The relationship between the thickness *h* and the volume of hydrogel precursor. Error bars denote the standard deviation of the measurements.

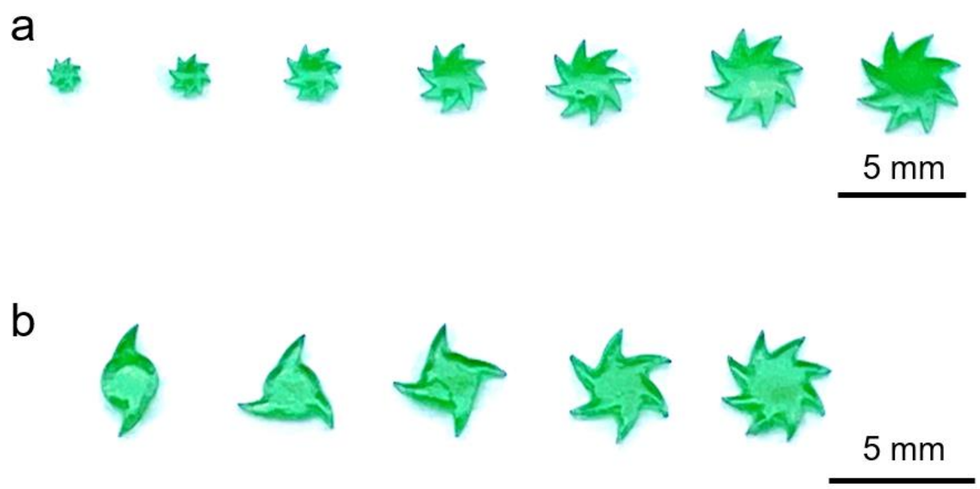

Supplementary Figure 6. Bio-inspired hydrogel rotors with different geometric parameters. (a) Rotors with *r* ranging from 250 µm to 1000 µm. (b) Rotors with teeth number *n* ranging from 2 to 8.

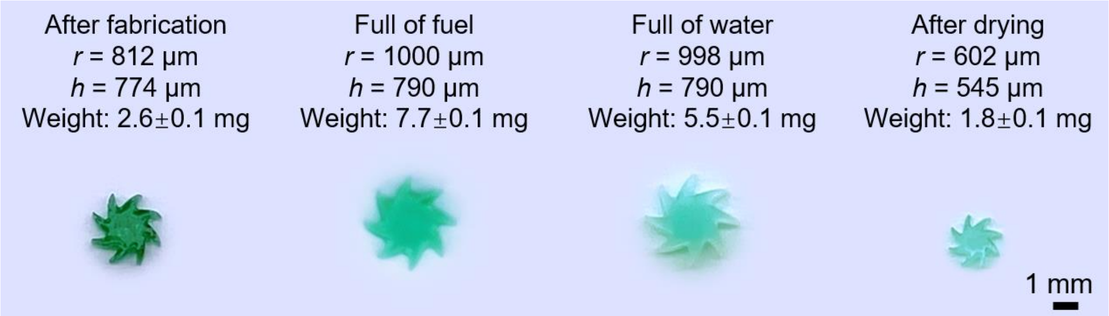

Supplementary Figure 7. The changes in shape and weight of hydrogel rotor under different states. By comparing the weight of hydrogel rotor when it is full of fuel and completely dried, we can obtain $w_{\mathrm{HFIP}}/w_{\mathrm{motor}}$ = 76.6%. By comparing the geometric sizes of hydrogel rotor when it is full of fuel and full of water, it can be demonstrated that PNIPAm has similar swelling behavior in HFIP and water. When the rotor is transferred into HFIP for fuel filling, some green dyes in the rotor diffuse into HFIP, which makes the color of the rotor fade.

Supplementary Note 1. Biocompatibility of HFIP fuel

HFIP is a metabolite of sevoflurane, which is approved by the Food and Drug Administration (FDA) and widely used in inhaled general anesthesia [1,2]. Approximately ~5% of the sevoflurane dose is metabolized into HFIP, which is then rapidly conjugated with glucuronic acid and excreted in urine [1]. In addition, some clinical studies have proved that intravenous administration of HFIP can reduce inflammation and improve survival in murine septic peritonitis [3,4]. According to the median lethal dose (LD_{50}, 0.18 mg/g in mice when administered intravenously, reported by U.S. Army Armament Research & Development Command, Chemical Systems Laboratory, NIOSH Exchange Chemicals. Vol. NX#03623), an adult mouse (~20 g) is injected with 3.6 mg of HFIP intravenously to achieve median lethality. The mass of HFIP in a certain rotor (r=250 µm, h=474 µm and c=8) is only ~0.2 mg, which is significantly below the intravenous lethal dose.

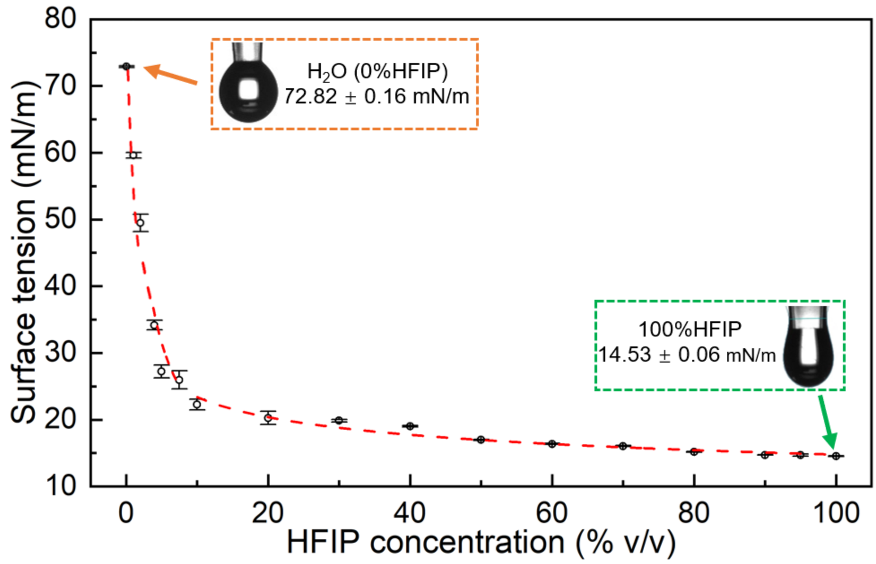

Supplementary Figure 8. Surface tension characterization of HFIP at different concentrations. The surface tension of HFIP was measured by optical contact angle measuring instrument (Theta Flex, Biolin Scientific, Finland) using the pendant drop method. As HFIP concentration increases from 0% to 100% (v/v), the surface tension decreases from 72.82 mN/m to 14.53 mN/m. Error bars denote the standard deviation of the measurements.

Supplementary Table 1: Different Chemical fuels in hydrogel motors. Density, surface tension, boiling point, maximum rotate speed, and maximum lifetime. The geometric parameters of hydrogel rotors for the test of maximum rotation speed: r=250 µm, h=474 µm and c=8. The geometric parameters of hydrogel rotors for the test of lifetime: r=1000 µm, h=790 µm and c=8.

Chemical fuel in hydrogel motors	Density (g/cm^3)	Surface tension (mN/m)	Boiling point(℃)	Maximum rotation speed(rpm)	Maximum lifetime(min)
N-propanol	0.804	23.78	97	2880±267	28.8±1.2
Isopropanol	0.786	21.70	82	1315±277	22.3±0.7
Ethanol	0.789	22.39	78	1284±83	10.1±1.0
Dimethyl sulfoxide	1.100	43.60	189	218±15	12.4±0.5
Acetone	0.790	26.26	56	436±81	6.0±0.3
**Hexafluoroisopropanol (HFIP)**	1.596	14.53	59	**5215±240**	**34.6±2.1**

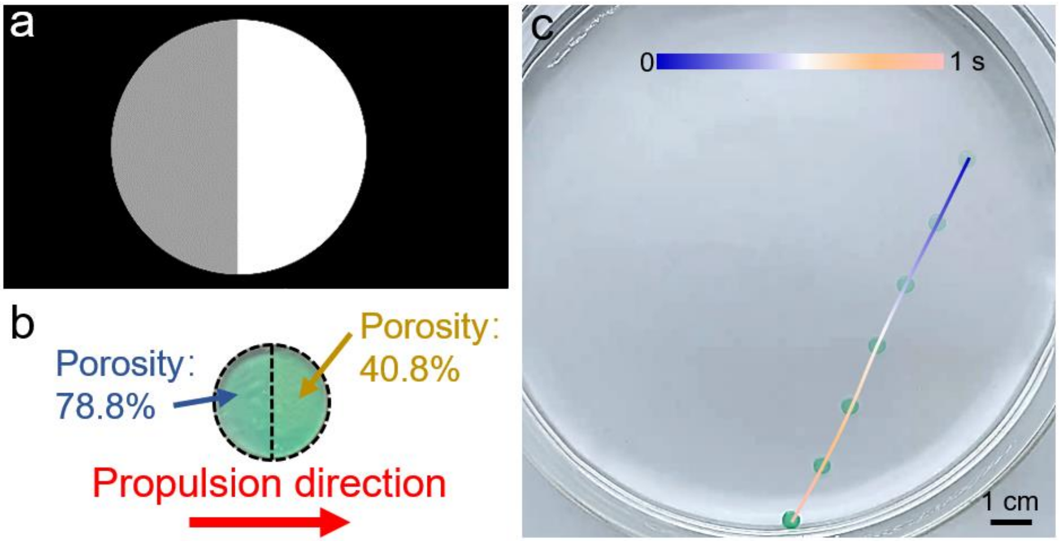

Supplementary Figure 9. Hydrogel disc (diameter of 4 mm, thickness of 2 mm) with asymmetric porosity. (a) The grayscale image used to process the asymmetric hydrogel disc. (b) Image of the hydrogel disc and schematic diagram of the propulsion direction on the water surface. (c) Time-lapse image of hydrogel disc moving on the water surface.

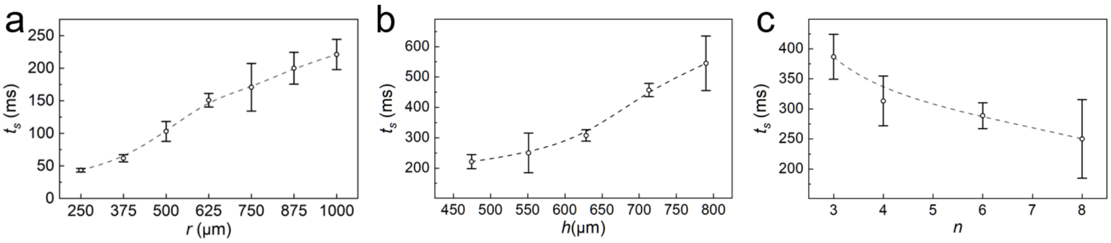

Supplementary Figure 10. The relationships between the time t_s to reach maximum rotation speed and geometric parameters of hydrogel rotor. The thickness *h* and teeth number *n* remain unchanged at 474 μm and 8 in (a), respectively. The radius *r* and teeth number *n* remain unchanged at 1000 μm and 8 in (b), respectively. The radius *r* and thickness *h* remain unchanged at 1000 μm and 551 μm in (c), respectively. Error bars denote the standard deviation of the measurements.

Supplementary Note 2. HFIP diffusion coefficients at different surface porosity

Through Einstein's equation of Brownian motion and semi-empirical pore-structure equations, the diffusion coefficient D_e of HFIP fuel in hydrogel can be expressed as [5,6]

$$D_e = \frac{D_w}{1 + c(1 - \varphi)}$$

where D_w is the diffusion coefficient of HFIP in water (3.2×10^{-9} m^2/s), φ is the surface porosity, and c is a structural parameter that depends on the cross section of the hydrogel. The pores in the cross section of the hydrogel can be approximately considered as circular (Supplementary Fig. S4), so $c = 2$ [6]. When the surface porosity of the hydrogel is 40.8% and 78.8%, the corresponding diffusion coefficients of HFIP are 1.47×10^{-9} m^2/s and 2.25×10^{-9} m^2/s, respectively.

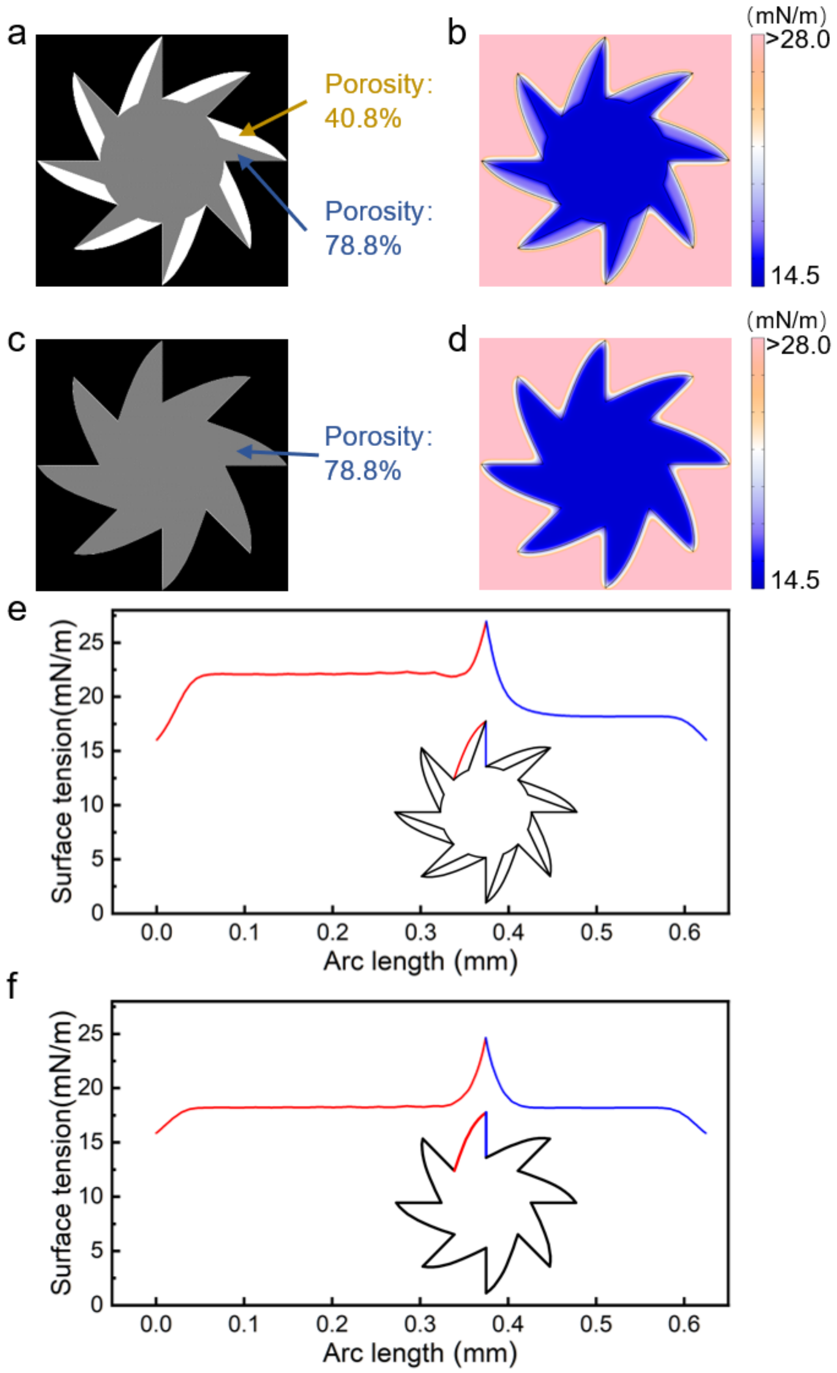

Supplementary Figure 11. Comparison between asymmetric rotor and symmetric rotor. (a) Surface porosity distribution of asymmetric rotor fabricated by grayscale digital light processing. (b) Simulation of surface tension distribution after asymmetric rotor is placed at the air-water interface for 43 ms. (c) Surface porosity of symmetric rotor. (d) Simulation of surface tension distribution after symmetric rotor is placed at the air-water interface for 43 ms. (e) Surface tension distribution along profile on one tooth of asymmetric rotor. Based on the simulation result, the surface tension torque M_s of asymmetric rotor is 1.62×10^{-9} N·m through numerical integration. (f) Surface tension distribution along profile on one tooth of symmetric rotor. Based on the simulation result, the surface tension torque M_s of symmetric rotor is 0.99×10^{-9} N·m through numerical integration.

Supplementary Table 2: Influence of rotor shape on surface tension distribution. Under the same type of curve, the curve shape is adjusted by changing parameters: Circular arc by changing radius, Spline curve by changing the position of control points, Parabola and Sine curve by changing the expression, Evolvent by changing the radius of the base circle.

Curve types		Suface tension distribution simulation (mN/m) at 43 ms	Surface tension distribution along profile on one tooth at 43 ms	M_s($\times 10^{-9}$ N·m) at 43 ms
1. Circular arc				
I_C	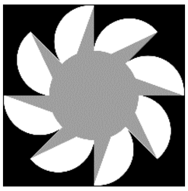	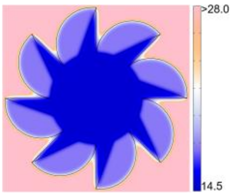	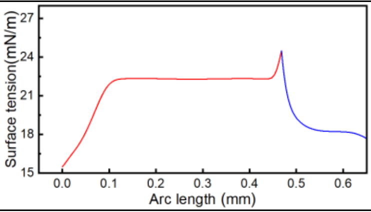	1.66
II_C	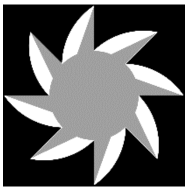	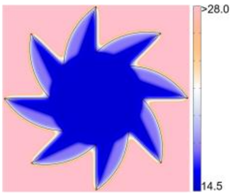	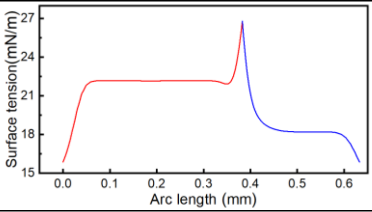	1.60
III_C	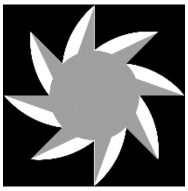	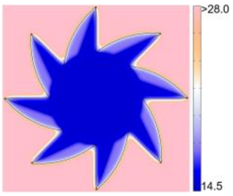	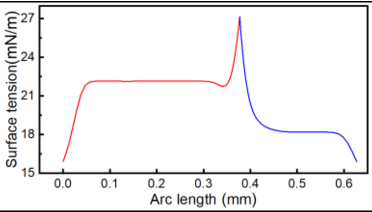	1.60
IV_C	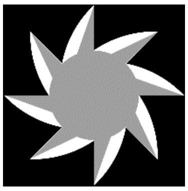	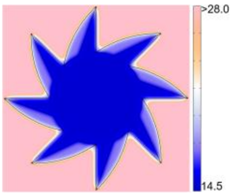	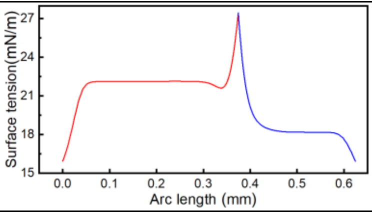	1.60
2. Spline curve				
I_{Sp}	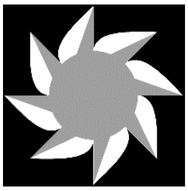	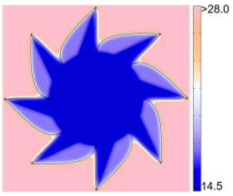	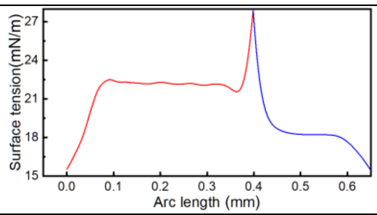	1.58
II_{Sp}	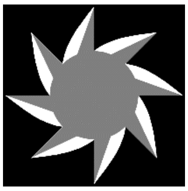	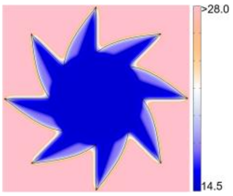	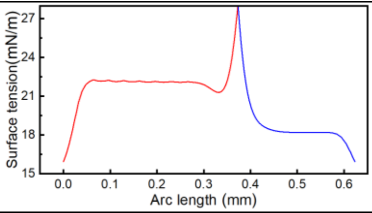	1.58

III_{Sp}	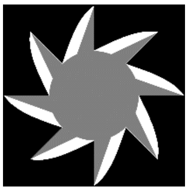	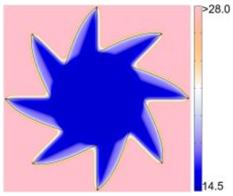	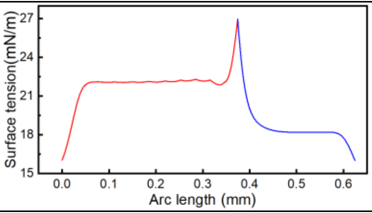	1.62
IV_{Sp}	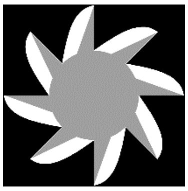	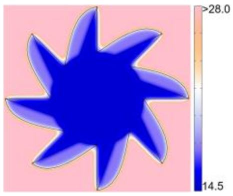	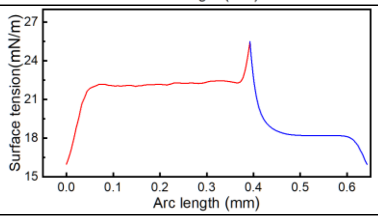	1.66
3. Parabola				
I_{P}	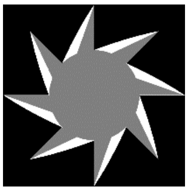	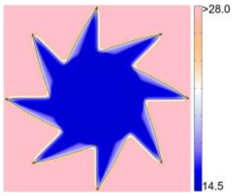	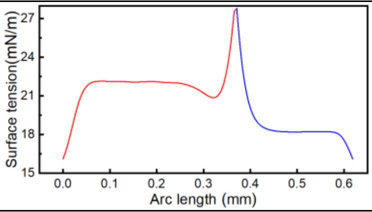	1.65
II_{P}	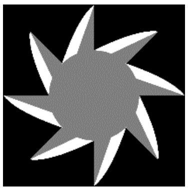	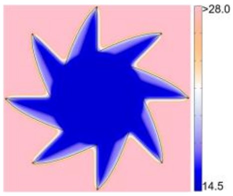	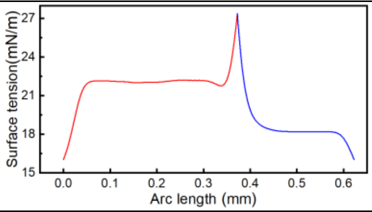	1.65
III_{P}	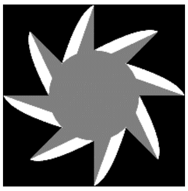	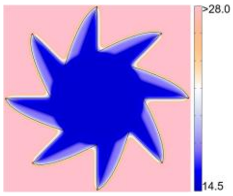	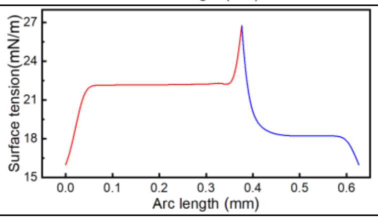	1.67
IV_{P}	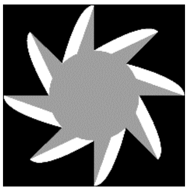	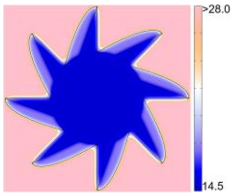	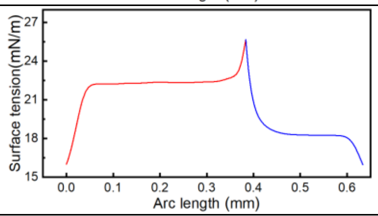	1.67
4. Sine curve				
I_{Si}	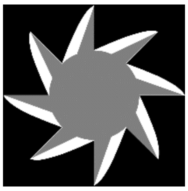	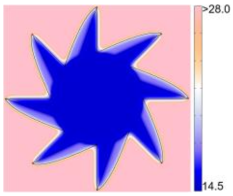	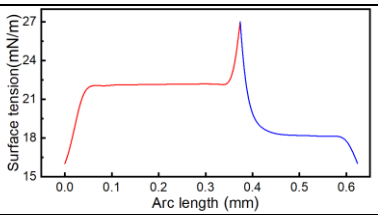	1.68
II_{Si}	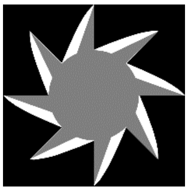	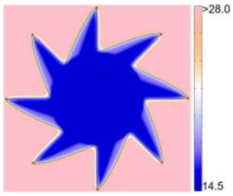	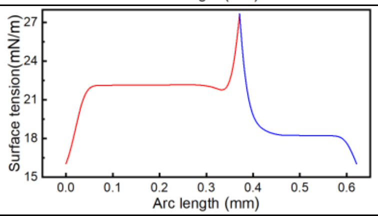	1.67

III_{Si}	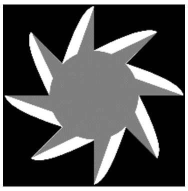	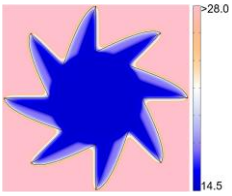	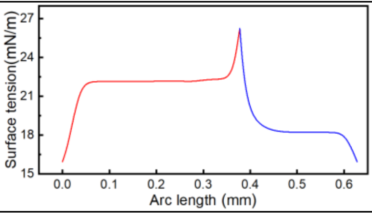	1.68
IV_{Si}	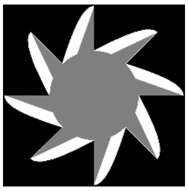	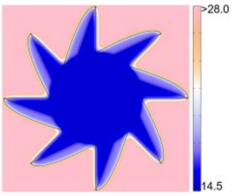	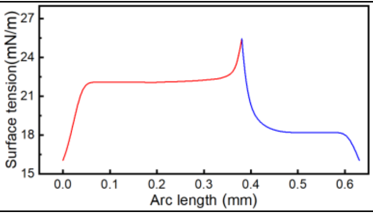	1.68
Type V: Evolvent				
I_E	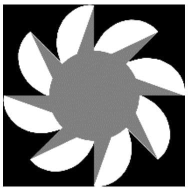	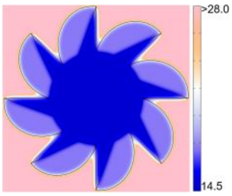	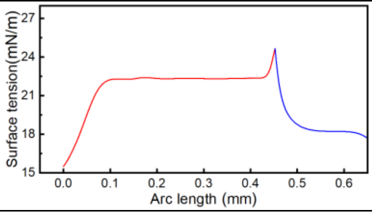	1.66
II_E	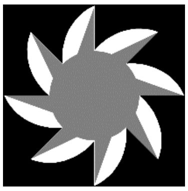	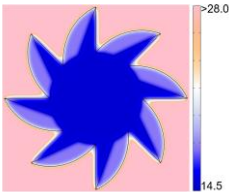	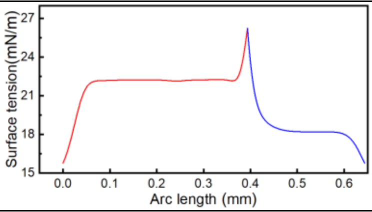	1.63
III_E	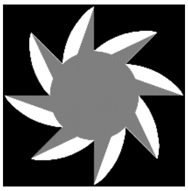	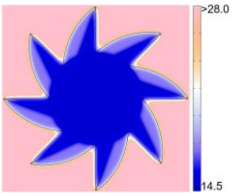	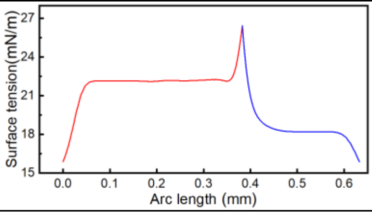	1.63
IV_E	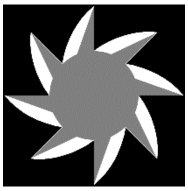	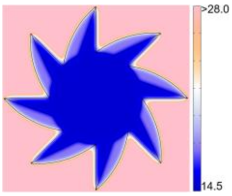	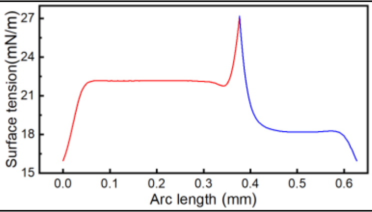	1.62

Supplementary Note 3. Method for evaluating drag reduction effect

For the influence of the shape on drag reduction in a specific flow field, the commonly used method in the industry is the combination of experiments and computational fluid dynamics (CFD). By keeping the flow field unchanged, the shape is modified through experiments and theoretical calculations to obtain the optimized drag reduction effect. This method is widely used in shape design of automobiles, airplanes, ships, missiles and so on [7-10]. We use this method to optimize the shape design of the rotor and realize drag reduction. First of all, a target rotation speed needs to be assumed to determine the flow field in order to continue the simulation and theoretical calculation, so that the theoretical resultant resistance torque under a specific rotor shape can be obtained. Herein, we specifically studied the theoretical resistance torques of the rotor at 5000 rpm. When the rotor rotates at the interface of water and air, it is mainly subjected to two resistances: the viscous resistance on the bottom surface and the pressure resistance on the side. Since the movement form of the rotor is rotation, it is necessary to further calculate the resistance torques (viscous resistance torque M_{v} and pressure resistance torque M_{p}) caused by these two resistances.

■ **Viscous resistance torque M_{v}**

If edge effect and the influence of any other boundaries are neglected, the resistance torque for one side of a disc is given by[11,12]

$$M_0 = \frac{0.308\pi\rho a^5\omega^2}{Re^{0.5}} \tag{S1}$$

where ρ is the density of the fluid, a is the radius of the disc, ω is the angular velocity of the disc and Re is the Reynolds number.The Reynolds number can be obtained by

$$Re = \frac{\omega a^2}{v} \tag{S2}$$

where v is the kinematic viscosity of the fluid. Because our rotor is not a complete disc, we introduce a coefficient η to modify Equation(S1) and obtain

$$M_v = \eta M_0 \tag{S3}$$

The coefficient η is related to the rotor shape. By numerical integration, coefficients η for different rotor shapes are obtained, as shown in Supplementary Table 3. According to geometric definition of the rotor shape (Fig. 1c), a is replaced by $2r$. Finally, the viscous resistance torque M_{v} of the rotor during the rotation can be evaluated by

$$M_v = 4.928\pi\eta\rho r^4\omega^{1.5}v^{0.5} \tag{S4}$$

■ **Pressure resistance torque M_{p}**

For pressure resistance torque, it is caused by the pressure of water on the submerged side wall of rotor during the rotation. Pressure resistance torque M_{p} can be evaluated by

$$M_p = \oint_L P \cdot h \cdot D_p \cdot cos\,\beta \cdot dl \tag{S5}$$

where L is the profile of rotor, P is pressure of water at differentiation element dl on rotor profile, h is the depth to which the rotor is immersed in water (approximately equal to the thickness of the rotor), D_{p} is the length from the differentiation element to

the rotor center, β is the angle between P and its component P'. P' is perpendicular to the straight line connected to the rotor center.

Based on the simulation results, we can get the resultant resistance torque M_r (sum of M_v and M_p) under different shapes by numerical integration. The smaller the resultant resistance torque, the better the drag reduction effect.

Supplementary Table 3: Influence of rotor shape on resistance torques. Under the same type of curve, the curve shape is adjusted by changing parameters: Type I: Circular arc by changing radius, Type II: Spline curve by changing the position of control points, Type III: Parabola and Type IV: Sine curve by changing the expression, Type V: Evolvent by changing the radius of the base circle.

Curve types		η	M_v(×10^{-9} N·m) at 5000 rpm	Pressure distribution simulation (Pa) at 5000 rpm	M_p(×10^{-9} N·m) at 5000 rpm	M_r(×10^{-9} N·m) at 5000 rpm
1. Circular arc						
I_C	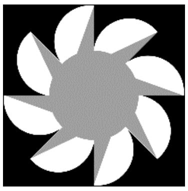	0.66	0.48	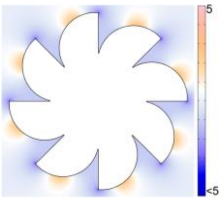	1.00	1.48
II_C	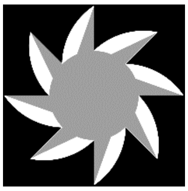	0.37	0.27	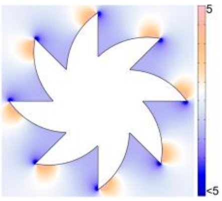	1.13	1.40
III_C	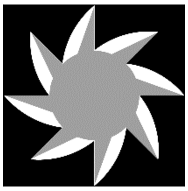	0.34	0.24	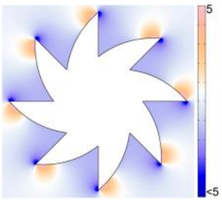	1.15	1.39
IV_C	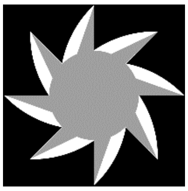	0.32	0.23	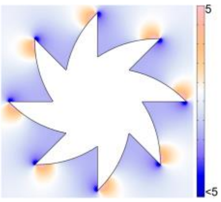	1.26	1.49
2. Spline curve						
I_{Sp}	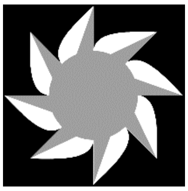	0.39	0.28	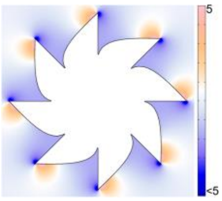	1.32	1.60
II_{Sp}	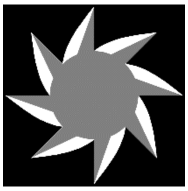	0.30	0.22	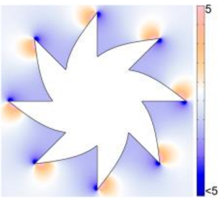	1.21	1.43

III_{Sp}	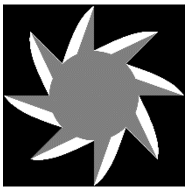	0.32	0.23	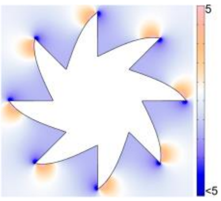	0.95	1.18
IV_{Sp}	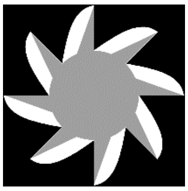	0.41	0.30	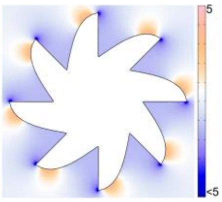	1.08	1.38
3. Parabola						
I_{P}	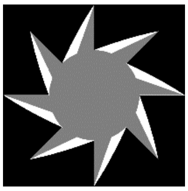	0.26	0.19	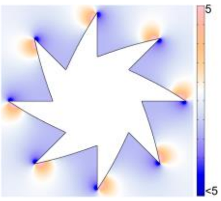	1.24	1.43
II_{P}	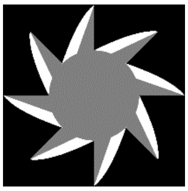	0.30	0.22	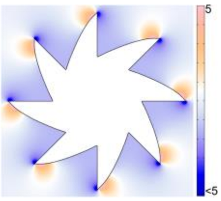	1.20	1.42
III_{P}	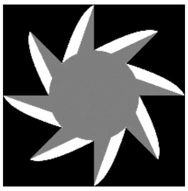	0.33	0.24	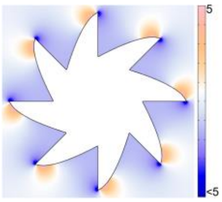	1.10	1.34
IV_{P}	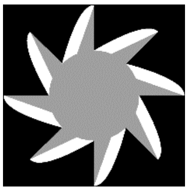	0.36	0.26	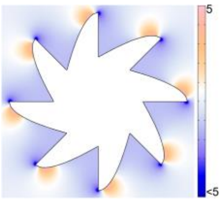	1.18	1.44
4. Sine curve						
I_{Si}	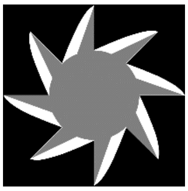	0.31	0.23	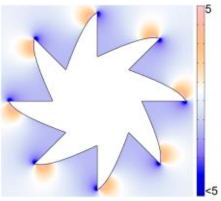	1.19	1.42
II_{Si}	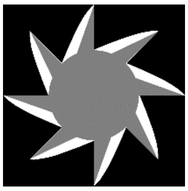	0.29	0.21	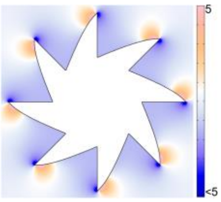	1.20	1.41

III_{Si}	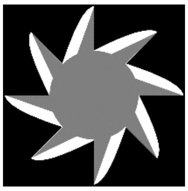	0.34	0.25	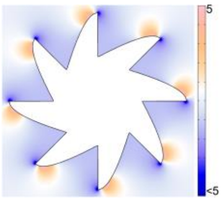	1.01	1.26
IV_{Si}	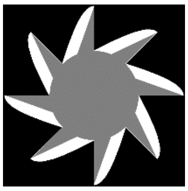	0.33	0.24	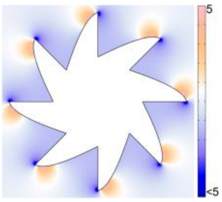	1.12	1.36
5. Evolvent						
I_E	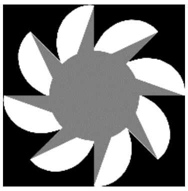	0.61	0.44	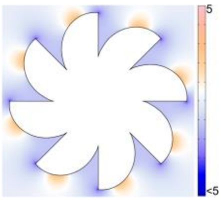	1.05	1.49
II_E	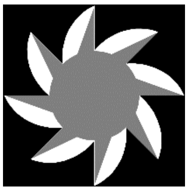	0.41	0.30	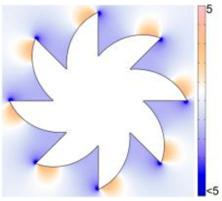	1.16	1.46
III_E	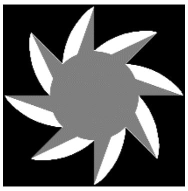	0.37	0.27	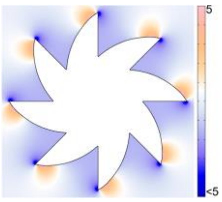	1.17	1.44
IV_E	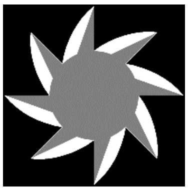	0.33	0.24	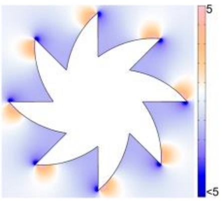	1.22	1.46

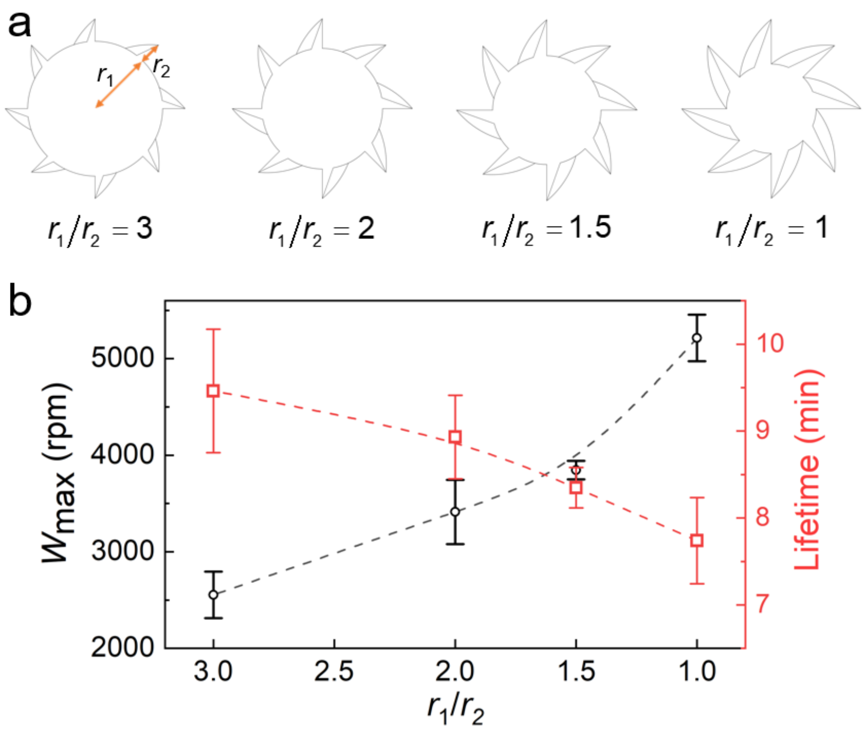

Supplementary Figure 12. Influence of the ratio (r_1/r_2) of inner circle radius to tooth length on rotor performance. The geometric parameters of the rotor are as follows: r=250 µm, h=474 µm and c=8. When the ratio decreases, the maximum rotation speed increases and the lifetime decreases. Compared to the increase of the maximum rotation speed, the decrease in lifetime is very small. Therefore, $r_1/r_2 = 1$ is chosen as the shape of the rotor. Error bars denote the standard deviation of the measurements.

Supplementary Table 4: Chemical Marangoni rotor benchmarking

Legend	Rotor type	Rotation output α_{max} (rpm/mm^3)	Fuel economy β_{max} (min/mg)	Reference in text
□	MOF rotors	734.48	7.81	[25]
□	MOF rotors	1.68	6.67	[26]
▽	polymer capsules	62.50	1.39	[17]
▽	polymer capsules	32.00	2.69	[18]
▽	polymer capsules	5.54	0.36	[19]
▽	polymer capsules	4.93	0.52	[35]
▽	polymer capsules	7.41	1.20	[30]
△	camphor boats	0.10	5.48	[34]
△	camphor boats	2.63	2.33	[31]
△	camphor boats	0.20	0.13	[33]
△	camphor boats	47.33	2.86	[32]
○	droplets/particles	15.85	5.95	[27]
○	droplets/particles	1830.00	29.41	[29]
○	droplets/particles	4.80	0.05	[28]
☆	bio-inspired hydrogel rotors	27175.15	39.33	This work

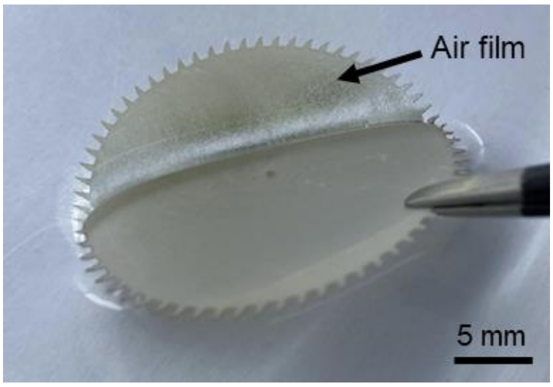

Supplementary Figure 13. The passive rotor is made of polystyrene film (200 μm thick) cut by femtosecond laser cutting. In addition, micro-nano structures are induced on the surface of the passive rotor by femtosecond laser ablation. The surface is then modified by a commercial superhydrophobic spray (Glaco Mirror Coat Zero, Soft 99 Ltd, Japan) to enhance the hydrophobicity. The air film formed between the water and the passive rotor can reduce friction.

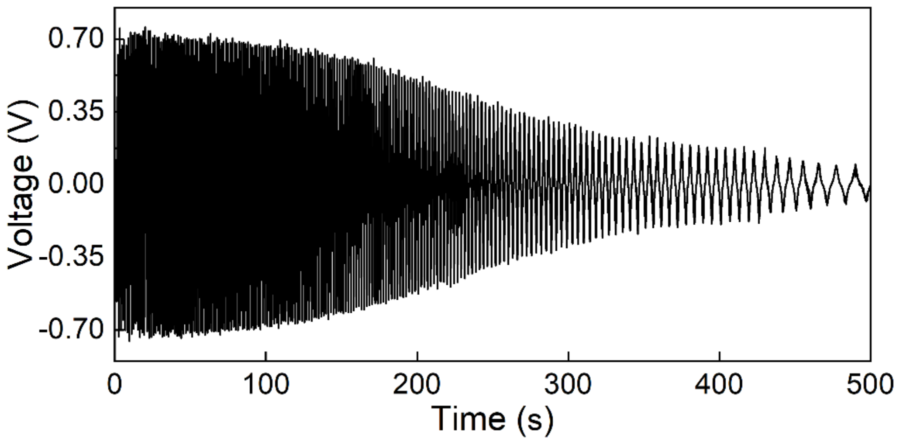

Supplementary Figure 14. Induced voltage generated by the mini-generator with time.

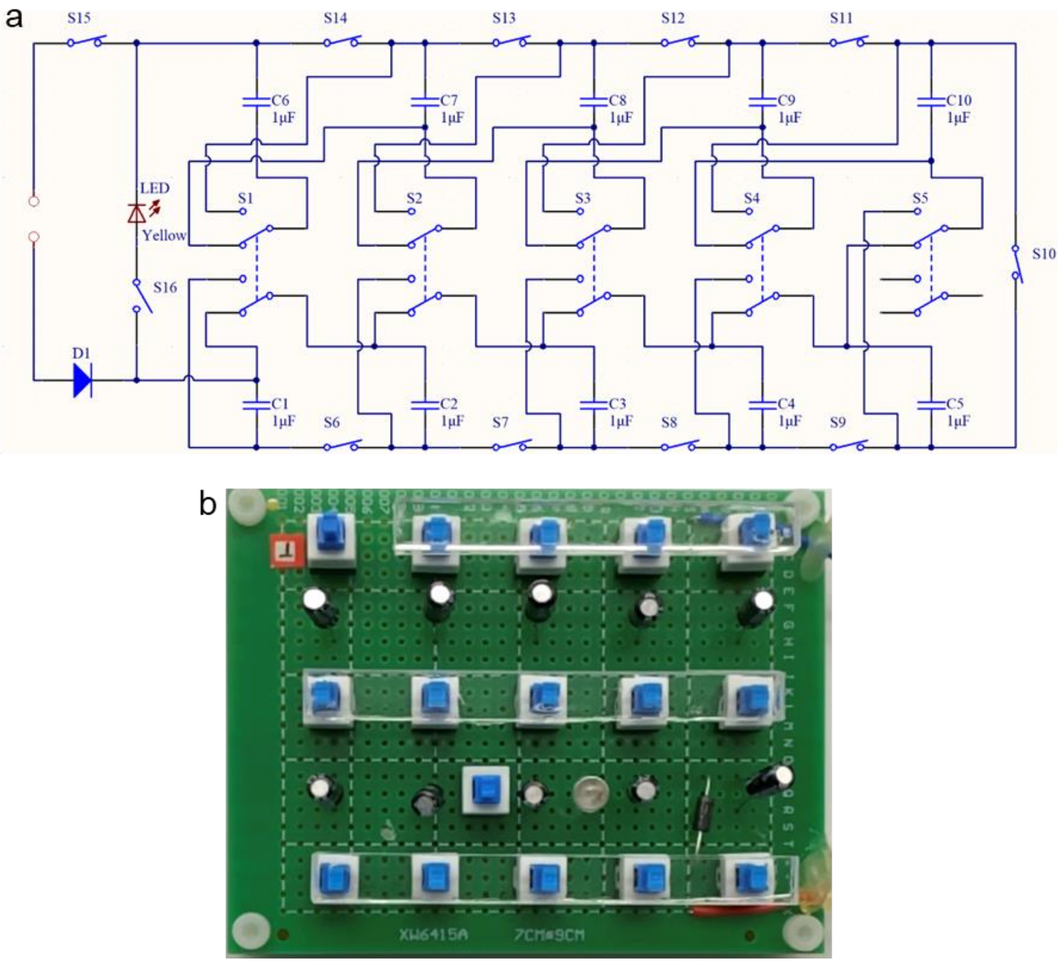

Supplementary Figure 15. Circuit schematic and board used to collect electrical energy and increase voltage. When collecting electric energy, ten capacitors are connected in parallel. When releasing electric energy, ten capacitors are switched to a serial manner to increase the voltage.

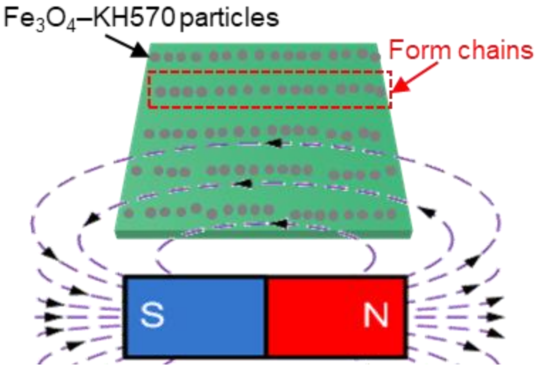

Supplementary Figure 16. Schematic diagram of the KH570-modified Fe_3O_4 nanoparticles in the hydrogel forming chains under a magnetic field. A magnet ($40\times40\times10$ mm^3) is placed under the hydrogel containing KH570-modified Fe_3O_4 nanoparticles and a horizontal magnetic field will form on the surface of the hydrogel. KH570-modified Fe_3O_4 nanoparticles are magnetized to assemble into chains along the magnetic field direction.

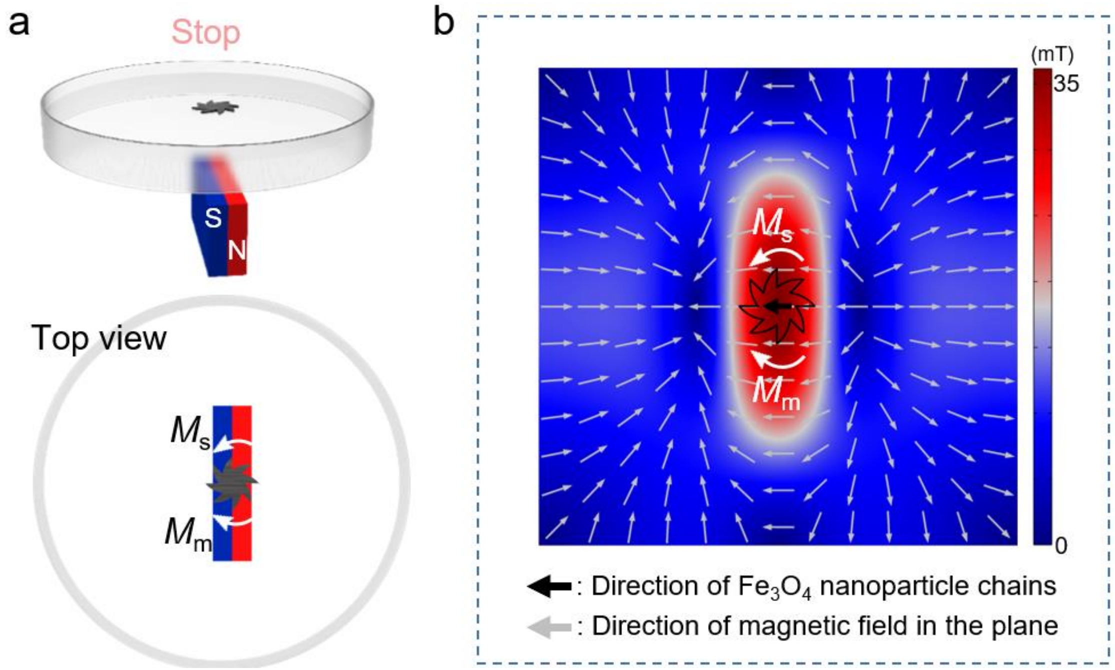

Supplementary Figure 17. (a) A cuboid magnet ($40\times40\times10$ mm^3) is placed 15 mm below the magnetic hydrogel rotor to switch it from rotation to stop. (b) Simulation of magnetic field formed in the plane of the rotor. The black arrow and gray arrows represent the direction of Fe_3O_4 nanoparticle chains and the direction of the magnetic field, respectively. Because the magnetic field region where the magnetic hydrogel rotor is located has almost no gradient, the rotor is not subjected to the force from the magnetic field. However, when the rotor rotates at a very little angle under the action of surface tension torque M_s (refer to Fig. 2 for details), the magnetic field has a magnetic torque M_m on the rotor because Fe_3O_4 nanoparticle chains inside the rotor tend to be consistent with the direction of magnetic induction line. The magnetic torque M_m and the surface tension torque M_s achieve a dynamic balance, so the rotor can be switched from rotation to stop under the magnetic field of the cuboid magnet. When the magnet is removed, the rotor resumes *in situ* rotation. The switching operation is repeatable and reversible.

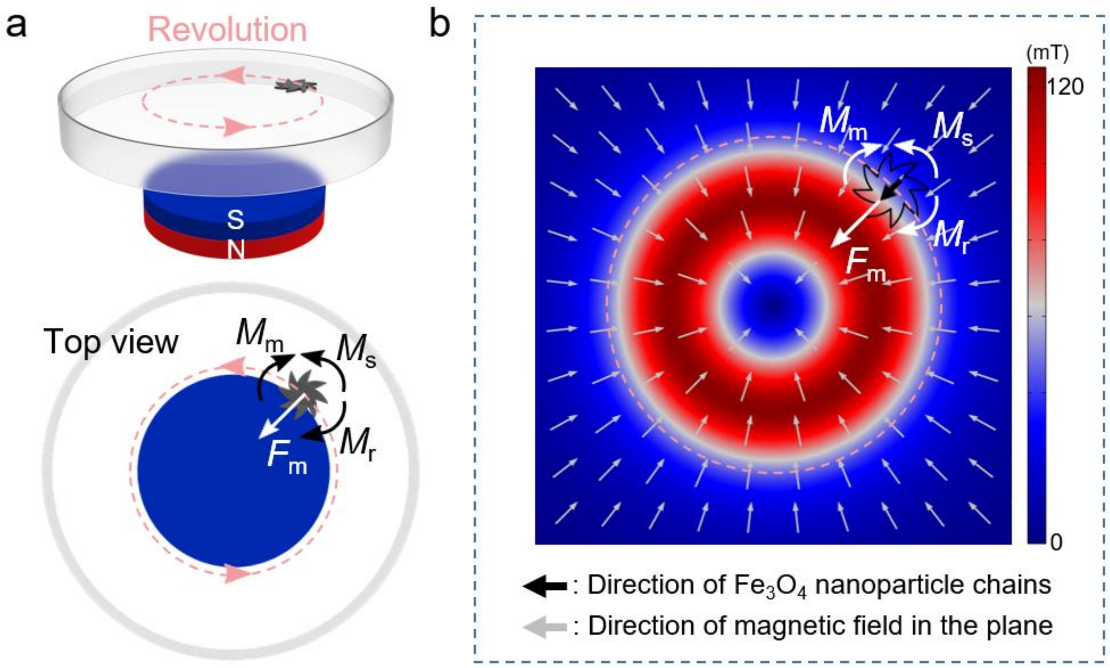

Supplementary Figure 18. (a) A cylindrical magnet (diameter of 50 mm, height of 10 mm) is placed 15 mm below the magnetic hydrogel rotor to switch it from rotation to revolution. (b) Simulation of magnetic field formed in the plane of the rotor. The black arrow and gray arrows represent the direction of Fe_3O_4 nanoparticle chains and the direction of the magnetic field, respectively. The magnetic hydrogel rotor is subjected to a force F_m towards the center due to the magnetic field gradient. Since Fe_3O_4 nanoparticle chains inside the rotor tend to be consistent with the direction of magnetic induction line, the rotor is also subjected to a torque M_m from the magnetic field during motion. In addition, the rotor is subjected to surface tension torque M_s and resistance torque M_r (refer to Fig. 2 for details). Among them, the force F_m contributes to the centripetal force required for the revolution of the rotor, and the three torques (M_m, M_s and M_r) contribute to the rotation of the rotor during revolution. Combined with the force and torques mentioned above, the magnetic hydrogel rotor can be switched from *in situ* rotation to revolution under the magnetic field of the cylindrical magnet. When the magnet is removed, the rotor resumes *in situ* rotation. The switching operation is repeatable and reversible.

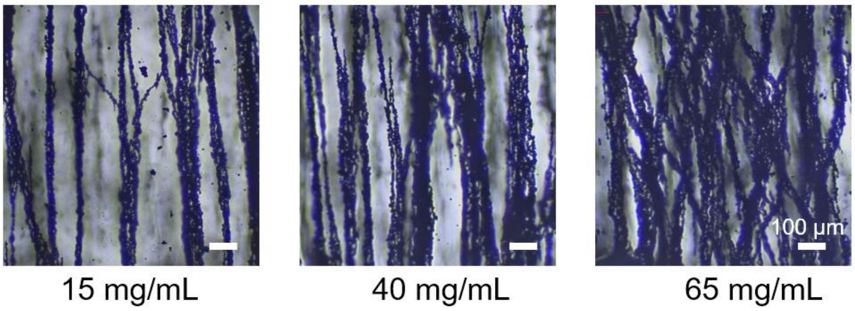

Supplementary Figure 19. Magnetic hydrogel rotors with three chain densities can be obtained by adding different doses of Fe_3O_4 nanoparticles in the hydrogel.

References

1. US Food & Drug Administration. *ULTANE® (sevoflurane) volatile liquid for inhalation* (2017).
2. Colomer, I., Chamberlain, A. E. R., Haughey, M. B. & Donohoe, T. J. Hexafluoroisopropanol as a highly versatile solvent. *Nat. Rev. Chem.* **1**, 0088 (2017).
3. Urner, M. et al. Insight into the beneficial immunomodulatory mechanism of the sevoflurane metabolite hexafluoro-2-propanol in a rat model of endotoxaemia. *Clin. & Exp. Immunol.* **181**, 468-479 (2015).
4. Herrmann, I. K. et al. Intravenous application of a primary sevoflurane metabolite improves outcome in murine septic peritonitis: first results. *PloS one* **8**, e72057 (2013).
5. Alsaid, Y. et al. Tunable Sponge-Like Hierarchically Porous Hydrogels with Simultaneously Enhanced Diffusivity and Mechanical Properties. *Adv. Mater.* **33**, 2008235 (2021).
6. Koponen, A., Kataja, M., & Timonen, J. Tortuous flow in porous media. *Phy. Rev. E* **54**, 406-410 (1996).
7. Sudin, M. N. et al. Review of research on vehicles aerodynamic drag reduction methods. *Intl J. Mech. Mechatron. Engng.* **14**, 35-47 (2014).
8. Yamazaki, W., Matsushima, K., & Nakahashi, K. Drag reduction of a near-sonic airplane by using computational fluid dynamics. *AIAA J* **43**, 1870-1877 (2005).
9. Shereena, S. G., Vengadesan, S., Idichandy, V. G. & Bhattacharyya, S. K. CFD study of drag reduction in axisymmetric underwater vehicles using air jets. *Eng. Appl. Comp. Fluid* **7**, 193-209 (2013).
10. Srinivasan, G. R. & Chamberlain, R. R. Drag reduction of spiked missile by heat addition. *AIAA Atmospheric Flight Mechanics Conference and Exhibit* 4714 (2004).
11. Goldstein, S. On the resistance to the rotation of a disc immersed in a fluid. *Math. Proc. Cambridge* **31**, 232-241 (1935).
12. Cochran, W. G. The flow due to a rotating disc. *Math. Proc. Cambridge* **30**, 365-375 (1934).
