# Supplementary material for: High-performance Marangoni hydrogel rotors with asymmetric porosity and drag reduction profile
Source: Nat Commun. 2023 Jan 3;14:20. doi: 10.1038/s41467-022-35186-5 (PMC9810638; doi:10.1038/s41467-022-35186-5)
Supplement: Supplementary file 2 — Description of Additional Supplementary Files [file 41467_2022_35186_MOESM2_ESM.pdf]

Description of Additional Supplementary Files

File Name: Supplementary Movie 1
Description: High-speed rotation of hydrogel rotor.

File Name: Supplementary Movie 2
Description: Long lifetime of hydrogel rotor

File Name: Supplementary Movie 3
Description: Rapid removal of water in rotor

File Name: Supplementary Movie 4
Description: Rapid fuel re-injection in rotor.

File Name: Supplementary Movie 5
Description: Kinetic energy transmission as speed reducer.

File Name: Supplementary Movie 6
Description: Kinetic energy transmission as speed multiplier.

File Name: Supplementary Movie 7
Description: Marangoni hydrogel rotors for mini-generators.

File Name: Supplementary Movie 8
Description: Generated electricity used to power LED bulb.

File Name: Supplementary Movie 9
Description: Control of magnetic hydrogel rotors: start-stop switching.

File Name: Supplementary Movie 10
Description: Control of magnetic hydrogel rotors: rotation and revolution switching.

File Name: Supplementary Movie 11
Description: Control of magnetic hydrogel rotors: revolving along the same orbit.

File Name: Supplementary Movie 12
Description: Control of magnetic hydrogel rotors: revolving along different orbits and directions.
